# Efficacy and Safety of Intra‐Articular Therapies for Temporomandibular Disorders: A Systematic Review and Meta‐Analysis

**DOI:** 10.1111/odi.70284

**Published:** 2026-03-12

**Authors:** Mateus Gaya dos Santos, Wellington Luiz de Oliveira da Rosa, Cristian Sbardelotto, Júlia Silva Gomes de Araújo, Camila da Silva Rodrigues, Noéli Boscato

**Affiliations:** ^1^ Graduate Program in Dentistry Federal University of Pelotas (UFPel) Pelotas Brazil; ^2^ Department of Dental Materials and Prosthodontics Institute of Science and Technology, São Paulo State University (UNESP) São José dos Campos Brazil

**Keywords:** adverse events, arthrocentesis, intra‐articular, temporomandibular disorder, temporomandibular joint

## Abstract

**Objective:**

This systematic review and meta‐analysis aimed to evaluate the most effective minimally invasive intra‐articular procedure for reducing pain and improving maximum mouth opening (MMO) in individuals diagnosed with painful articular TMD, specifically TMJ osteoarthritis and/or internal derangement.

**Materials and Methods:**

Only randomized clinical trials (RCTs) assessing the efficacy of minimally invasive procedures, such as arthrocentesis and intra‐articular injection (IAI), were included. The meta‐analysis included seven studies with a 6‐month follow‐up, while 23 studies with different follow‐up periods were evaluated qualitatively.

**Results:**

The meta‐analysis showed that IAI with tenoxicam was the most effective procedure for pain reduction, with the highest [7.44 mean difference (MD); 6.28–8.60 confidence interval (CI)], followed by opioids (5.93; 5.03–6.83), the combination of hyaluronic acid (HA) with platelet‐rich plasma (PRP) (5.10; 4.52–5.68), and PRP alone (4.99; 3.13–6.85). For improvement in MMO, tenoxicam had the highest MD (+11.50 mm; 15.47–7.53), followed by PRP (+10.46 mm; 14.89–6.02) and the HA‐PRP combination (+10.10 mm; 11.89–8.31).

**Conclusions:**

Tenoxicam showed promising results for pain reduction and improvement in MMO, although the evidence remains limited, followed by opioids and the combination of HA and PRP with arthrocentesis. Further high‐quality studies are needed to confirm their clinical applicability.

## 1 Introduction

Temporomandibular disorders (TMDs) are a major cause of non‐dental orofacial pain (Schiffman et al. 2014; Valesan et al. 2021). These disorders affect the masticatory muscles, temporomandibular joint (TMJ), and/or related structures (Conti et al. 2020). TMD impacts approximately 31% of adults and 11% of children (Valesan et al. 2021) and is categorized into two subgroups: articular (TMJ‐related) and muscular (stomatognathic musculature) TMDs (Chang et al. 2018).

The TMJ consists of the mandibular condyle, the mandibular fossa of the temporal bone, and a fibrocartilaginous disc, enabling essential functions such as chewing and speaking (Oliveira et al. 2023). Despite being smaller than other joints, the TMJ is a complex structure that makes it susceptible to disorders that can result in pain and functional limitations (Alhaj Kheder et al. 2024; Ozsari et al. 2023), common symptoms of TMD (Alhaj Kheder et al. 2024; Durham et al. 2015). The Diagnostic Criteria for Temporomandibular Disorders (DC/TMD) is a diagnostic tool that identifies 12 subtypes of TMD using two axes (Schiffman et al. 2014). Wilkes' classification categorizes TMJ derangements into five stages, from early changes to severe joint destruction (Wilkes 1989). Besides, the Helkimo Index (HI) assesses dysfunction through three indices: anamnesis, clinical dysfunction, and occlusion (Helkimo 1974).

TMD typically involves initial management with conservative (e.g., occlusal splints) and minimally invasive (e.g., arthrocentesis and intra‐articular injections [IAI]) procedures, while surgical interventions are generally reserved for severe articular cases (Rodhen et al. 2022) for patients who do not respond to any initial treatments (Rodhen et al. 2022). Arthrocentesis is considered a cost‐effective and relatively quick procedure (Alhaj Kheder et al. 2024). Saline, Ringer's lactate (RL), hyaluronic acid (HA), and platelet‐rich plasma (PRP) are common IAI used in arthrocentesis or viscosupplementation (Bergstrand et al. 2019; Huddleston Slater et al. 2012). IAI helps flush and lubricate the TMJ, promote tissue healing (Asadpour et al. 2022), reduce pain (Asadpour et al. 2022; Bertolami et al. 1993; Hepguler et al. 2002), and enhance joint function (Alhaj Kheder et al. 2024; Bonotto et al. 2011). Biosupplementation, which relies exclusively on autologous products like PRP, supports tissue regeneration and modulates inflammation (Asadpour et al. 2022; Cömert Kılıç 2021).

IAI used in the management of TMD exhibits distinct characteristics and mechanisms of action, which directly influence clinical outcomes. Understanding these specificities is essential to optimizing treatment and addressing the individual needs of each patient (Alhaj Kheder et al. 2024). Despite the availability of various IAI types, the lack of clear clinical guidelines on which option is most effective for pain relief and improvement in mandibular function contributes to uncertainty in therapeutic decision‐making. Although multiple IAI are currently used, scientific evidence supporting the superiority of one over another remains inconclusive or inconsistent (Alhaj Kheder et al. 2024; Huddleston Slater et al. 2012).

Therefore, this systematic review with meta‐analysis of randomized controlled trials (RCTs) aimed to evaluate the most effective minimally invasive intra‐articular procedure for reducing TMJ pain and improving maximum mouth opening (MMO) in individuals diagnosed with painful articular TMD, *specifically TMJ* osteoarthritis and/or internal derangement. Safety and tolerability were also assessed based on reported adverse effects.

## 2 Material and Methods

### 2.1 Protocol and Registration

This systematic review was conducted following the guidelines outlined in the Cochrane Handbook for Systematic Reviews of Interventions and is reported following the Preferred Reporting Items for Systematic Reviews and Meta‐Analyses (PRISMA) guidelines (Page et al. 2022). The review protocol was registered in the Open Science Framework (OSF) database (osf.io/m9d2e).

The research question was developed using the PICO framework: *Population*: individuals diagnosed with painful articular TMD, specifically TMJ osteoarthritis and/or internal derangement; *Intervention*: minimally invasive TMJ procedures related to any IAI performed either alone or in combination with arthrocentesis; *Comparison*: pre–(baseline, T0) and post‐treatment within‐group comparisons; *Outcome*: pain intensity, using the Visual Analogue Scale (VAS), and improvement in MMO. Adverse events were also collected when reported.

Thus, the following research *question* was formulated: “What is the most effective minimally invasive intra‐articular procedure for reducing pain and improving MMO in individuals with painful articular TMD, when comparing IAI pre‐ and post‐treatment?”

### 2.2 Information Sources and Search Strategy

The search was carried out by two independent reviewers (J.A. and M.G.) across five databases, Medline/PubMed, Scopus, Embase (Elsevier), Web of Science, and Virtual Health Library: VHL (BIREME) ‐ Portal Regional, following each syntax rule as shown in Table S1. The last search was performed on the 16th of September 2024. Additionally, the gray literature (ProQuest) and the references of the studies included were also searched. No language restriction was applied.

### 2.3 Eligibility Criteria

Randomized clinical trials (RCTs) assessing patients diagnosed with osteoarthritis and/or TMJ internal derangement who received arthrocentesis alone or in combination with any IAI into the TMJ for TMD management were included. The diagnosis of osteoarthritis and/or TMJ internal derangement must be performed according to the DC/TMD criteria, Wilkes, HI, or Hegab classification, using any IAI alone or combined with arthrocentesis for the management of articular TMD. Intragroup (i.e., before and 6‐months after treatment) and between‐group comparisons (i.e., after treatment) were made.

Literature reviews, case reports, case–control and retrospective studies, systematic and narrative reviews, and studies that did not provide information on MMO and pain assessment using VAS scores or have a follow‐up period of less than 1 month were excluded.

### 2.4 Data Management

Studies that aligned with the inclusion criteria but lacked sufficient information in the title or abstract for a definitive decision were moved forward for full‐text review. In cases where duplicate studies or overlapping samples were identified, preference was given to the version with either a longer follow‐up duration or the most recent publication. After full‐text analysis, those that fulfilled the eligibility requirements were included in the data extraction stage. Additionally, reference lists of all qualifying studies were manually reviewed to minimize the risk of overlooking relevant publications. Any disagreements about study eligibility were resolved through discussion and consensus by a third reviewer (N.B.).

Data were extracted using a standardized form in Microsoft Office Excel 2016 (Microsoft Corporation, Redmond, WA, USA), covering the following key information for all studies: title, authors, publication year, adverse events, inclusion criteria, population, sample size, number of female and male participants, participant age, TMD diagnostic method, intervention, viscous solution used, number of applications, and time intervals for outcome assessments based on VAS and MMO at a 6‐months follow‐up. When reported, the number of IAIs administered was also recorded. For missing or unpublished data, authors were contacted via email, with up to three attempts at 7‐day intervals, and studies were excluded if data were not provided.

### 2.5 Risk of Bias

Two reviewers independently evaluated the risk of bias in each study. For randomized clinical trials, the RoB‐2 tool was utilized, and studies were categorized as having “low risk,” “some concerns,” or “high risk” of bias.

### 2.6 Meta‐Analyses

The quantitative analysis focused exclusively on VAS and MMO data, while outcomes presented as scores, percentages, or descriptive results were included in the qualitative synthesis. Sensitivity analyses were conducted to assess the robustness of pooled estimates, particularly after excluding non‐randomized trials or studies identified as having a high risk of bias. Additional sensitivity assessments accounted for missing post‐treatment data, interpreting these as either treatment success or failure scenarios.

Meta‐analyses were performed using Review Manager version 5.2 (The Nordic Cochrane Center, The Cochrane Collaboration, Copenhagen, Denmark) for studies that shared comparable control groups and reported identical outcome variables (VAS and MMO). A random‐effects model was applied for the overall analysis. Statistical significance was defined as a *p*‐value less than 0.05.

Variability in treatment effects across studies was evaluated using Cochran's Q test along with the *I* ^2^ statistic to assess heterogeneity. Additionally, publication bias for the VAS and MMO outcomes was assessed through visual inspection of the funnel plot and Egger's test, conducted in R (version 4.5.1; R Core Team, Austria).

In clinical practice, the change experienced by patients after treatment is often the primary indicator of effectiveness. Given the variability in baseline pain and MMO values across patients, the most appropriate approach to assess treatment effectiveness was to calculate the mean difference between pre‐ and postoperative values within the same group. Thus, combined effect estimates for mean differences in VAS and MMO, along with their standard deviations (SD), were computed both within groups (pre‐ and post‐treatment) and between groups following different interventions.

The data were categorized based on follow‐up periods: from baseline up to 6 months, given the limited number of clinical studies with a 6‐month follow‐up and considering that it is the optimal period for assessing these procedure efficacies (Bertolami et al. 1993), only studies with a 6‐month follow‐up were included in the meta‐analysis. This time frame was prioritized as it allowed for a broader inclusion of tested substances, facilitating a more comprehensive evaluation of therapeutic effects and intervention efficacy.

Intragroup comparisons (pre‐ and post‐treatment) were performed for VAS and MMO outcomes, considering baseline (T0) and 6‐month post‐intervention (T6) comparisons. The studies were categorized into subgroups according to the IAI used. Meta‐analysis also compared VAS scores from studies using one or two HA IAI. RCTs reporting MMO and VAS outcomes outside a 6‐month follow‐up were included in the qualitative analysis. When reported, adverse events were gathered.

### 2.7 Quality of Evidence

Considering the Quality of Evidence—GRADE Assessment was used to assess the confidence in the cumulative evidence. The GRADE (Grading of Recommendations, Assessment, Development, and Evaluations) approach was used, focusing on the outcomes VAS and MMO. The assessment was conducted using the GRADEpro Guideline Development Tool (GRADEpro GDT, McMaster University and Evidence Prime, 2022).

## 3 Results

**FIGURE 1:**
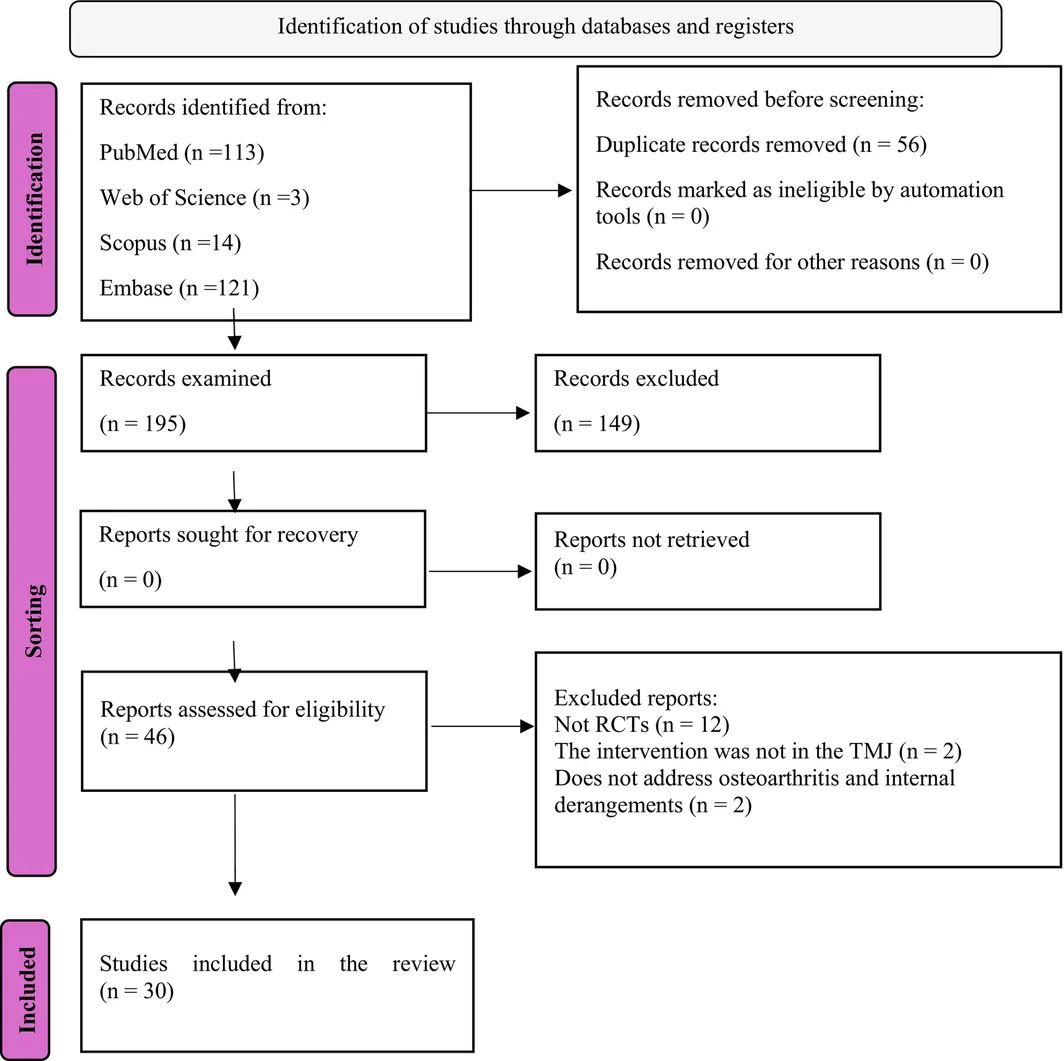
Flow chart illustrating the identification, screening, and inclusion of studies using databases and registers, as per PRISMA 2020.

The search strategy initially identified 251 studies, with 56 duplicates removed (Figure 1). Forty‐six studies underwent full‐text assessments, and 16 were excluded for not meeting the eligibility criteria. Thirty RCTs published between 1993 and 2024 assessed patients diagnosed with TMJ disorders using DC/TMD criteria, Wilkes, HI, or Hegab classification, and participants had previously failed conservative treatments. Thirty studies were included, and a total of seven RCTs provided data for the meta‐analysis (Table 1).

**TABLE 1:** Main characteristics of the studies included in the meta‐analysis.

Author	Follow‐up time	VAS assessed	MMO assessed	Total participants	Evaluated groups	Participants per group
Alhaj Kheder et al. (2024)	6 months	Y	N	22	Group: Non‐ultrasound‐guided 10% Dextrose injection	11
					Group: Ultrasound‐guided 10% Dextrose injection	11
Asadpour et al. (2022)	6 months	Y	Y	30	Group A: Arthrocentesis with RL + HA	10
					Group B: Arthrocentesis with RL + PRP	10
					Group C: Arthrocentesis with RL + HA combined with PRP	10
Bayramoglu et al. (2023)	6 months	Y	Y	30	Control Group: Arthrocentesis RL	14
					TX Group: Arthrocentesis RL + Tenoxicam	16
Dasukil et al. (2022)	6 months	Y	Y	90	Group A: Arthrocentesis with RL	30
					Group B: HA after Arthrocentesis with RL	30
					Group C: PRP after Arthrocentesis with RL	30
Korkmaz et al. (2016)	6 months	Y	Y	51	Group 1: Control; No treatment	13
					Group 2: Single HA injection	13
					Group 3: Double HA injection	13
					Group 4: Stabilization (occlusal) splint	12
Patel et al. (2016)	6 months	Y	Y	30	Group 1: Arthrocentesis with RL	15
					Group 2: Arthrocentesis with RL + HA	15
Sipahi et al. (2015)	6 months	Y	Y	30	Group 1: Arthrocentesis with RL	10
					Group 2: Arthrocentesis with RL + Morphine	10
					Group 3: Arthrocentesis with RL + Tramadol	10

Abbreviations: HA, hyaluronic acid; MMO, maximum mouth opening; N, not evaluated; PRP, platelet‐rich plasma; RL, Ringer's lactate; VAS, visual analog scale; Y, evaluated.

The remaining 23 RCTs were analyzed qualitatively due to non‐comparable data (Supporting Information S2 for VAS and Supporting Information S3 for MMO).

### 3.1 Risk of Bias

**FIGURE 2:**
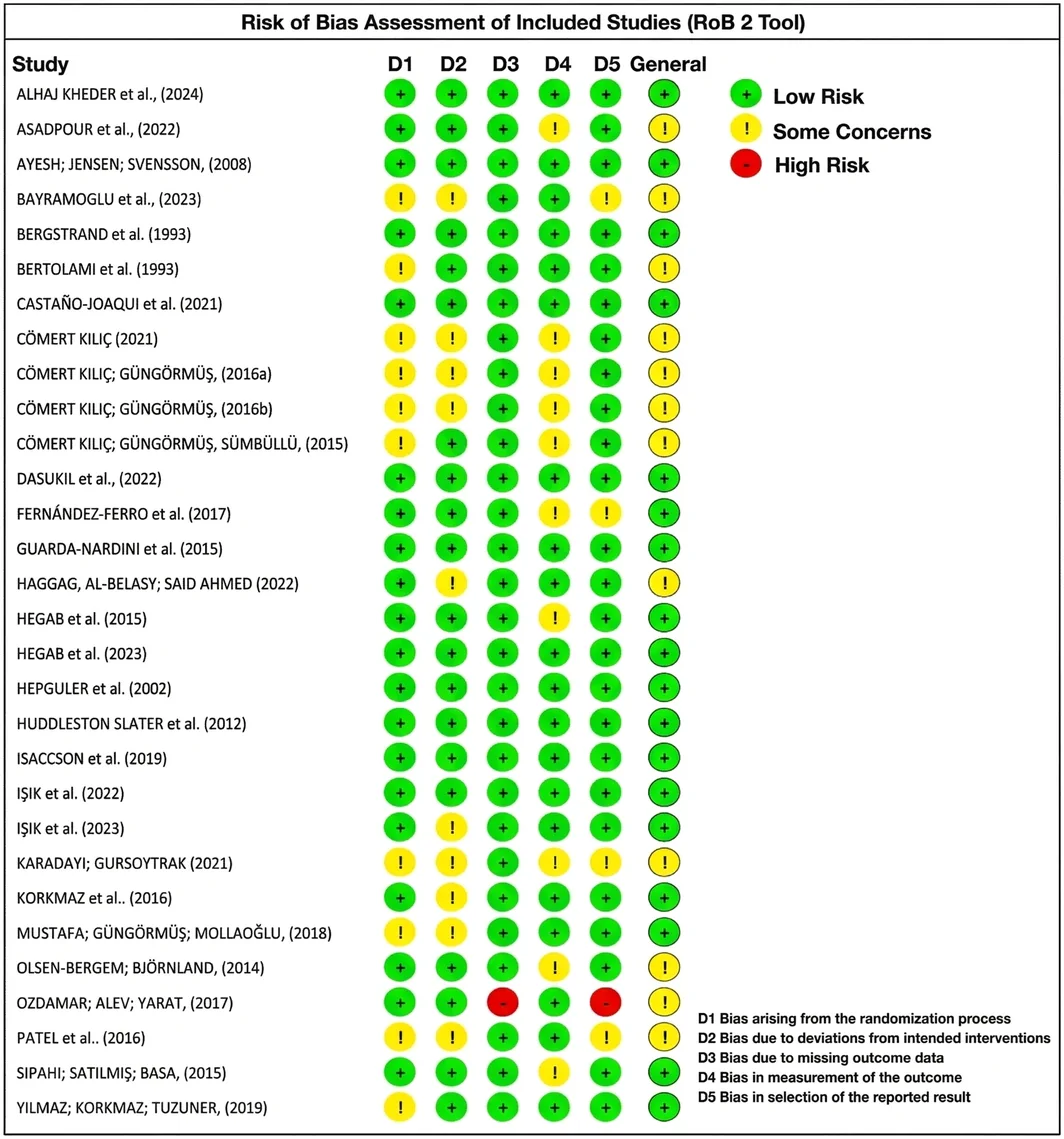
Risk of bias classification using RoB 2 for all RCTs included in this systematic review.

Regarding the *risk of bias*, 60.0% of the RCTs were classified as having a low risk of bias, while 40.0% raised some concerns and were categorized as having a moderate risk of bias (Asadpour et al. 2022; Bertolami et al. 1993; Cömert Kılıç 2021; Bayramoglu et al. 2023; Patel et al. 2016; Cömert Kılıç and Güngörmüş 2016a; Ozdamar et al. 2017). Notably, no study exhibited an elevated risk of bias based on the evaluated criteria (Figure 2).

### 3.2 Meta‐Analysis

**FIGURE 3:**
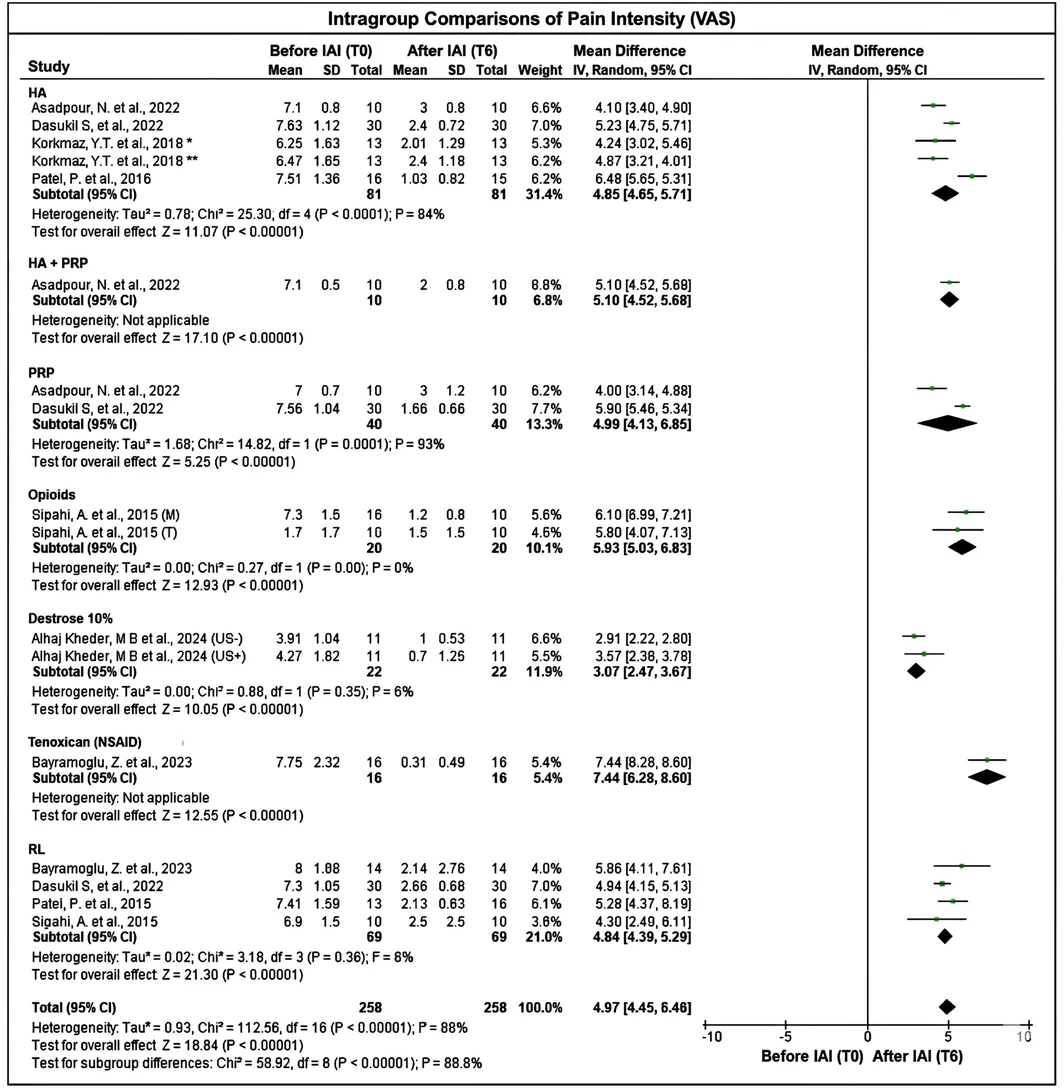
Meta‐analysis of intragroup comparisons of pain intensity measured using the Visual Analog Scale (VAS) at baseline (T0) and 6 months after (T6) arthrocentesis and/or intra‐articular injection (IAI) procedures. All substances tested, whether natural or synthetic, significantly reduced pain intensity after the intervention (*p* < 0.05), regardless of the specific substance used. (*), one injection of HA; (**), two injections of HA; CI, confidence interval; HA + PRP, hyaluronic acid combined with platelet‐rich plasma; HA, hyaluronic acid; IAI, intra‐articular injection; NSAID, tenoxicam; opioids, tramadol (T) and morphine (M); PRP, platelet‐rich plasma; RL, Ringer's lactate; T0, baseline; T6, 6 months after the IAI.

**FIGURE 4:**
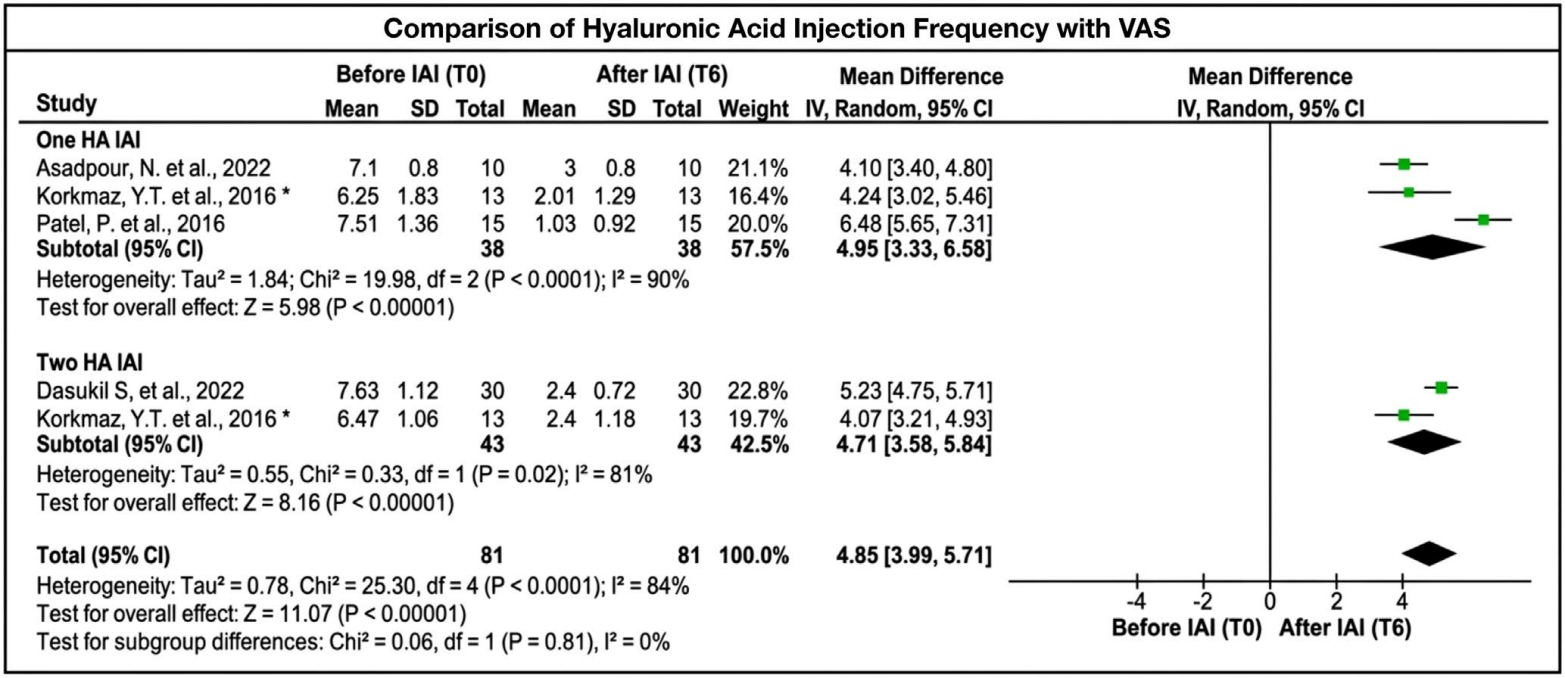
Meta‐analysis comparing the number of hyaluronic acid (HA) intra‐articular injections (IAIs) based on pain intensity measured using the Visual Analog Scale (VAS). Mean differences in pain reduction are shown according to the number of HA IAIs administered. CI, confidence interval; HA, hyaluronic acid; IAI, intra‐articular injection; VAS, Visual Analog Scale.

In the studies included in the meta‐analysis, 71% of the participants were women (*n* = 201), with an average age of 32 years (standard deviation of ±5.76 years). In the pain intragroup comparisons, pre‐ and 6‐months post‐IAI, seven studies reported a significant reduction in VAS scores (Alhaj Kheder et al. 2024; Asadpour et al. 2022; Bayramoglu et al. 2023; Dasukil et al. 2022; Korkmaz et al. 2016; Patel et al. 2016; Sipahi et al. 2015) and five an improvement in MMO values (Asadpour et al. 2022; Bayramoglu et al. 2023; Dasukil et al. 2022; Korkmaz et al. 2016; Patel et al. 2016) (Figures 3 and 4). HA (Asadpour et al. 2022; Dasukil et al. 2022; Korkmaz et al. 2016; Patel et al. 2016), RL (Bayramoglu et al. 2023; Dasukil et al. 2022; Korkmaz et al. 2016; Patel et al. 2016; Sipahi et al. 2015), PRP (Asadpour et al. 2022; Dasukil et al. 2022), dextrose 10% (Alhaj Kheder et al. 2024), HA combined with PRP (Asadpour et al. 2022), NSAID (Bayramoglu et al. 2023), opioids, morphine, and tramadol (Sipahi et al. 2015), regardless of the number of IAI. Adverse effects are described in Table 2.

**TABLE 2:** Adverse events reported in the studies included in the systematic review.

Author	Adverse events
Alhaj Kheder et al. (2024)	No post‐procedure complications were reported in either group.
Asadpour et al. (2022)	No complications were observed in our patients.
Ayesh et al. (2008)	None of the patients in this study reported any side effects or systemic psychomimetic symptoms after the injections.
Bayramoglu et al. (2023)	Not reported.
Bergstrand et al. (2019)	No patient developed other complications except for transient facial nerve paresis due to the use of local anesthesia.
Bertolami et al. (1993)	There were 13 adverse events in 10 patients, most of which were mild and short‐lived. Seven events occurred in six patients with hyaluronate, and six in four with placebo, usually lasting 1 day. In the hyaluronate group, five events are mild, one moderate, and one severe; in the placebo group, four were mild and two were severe. All were resolved, suggesting that hyaluronate injection in the TMJ is safe and comparable to placebo in terms of adverse event risk.
Castaño‐Joaqui et al. (2021)	A total of 20 adverse events were recorded. The most common was irrigation fluid extravasation (13 cases). There were two cases of intraoperative bleeding, three of facial paralysis, one of impaired vision, and one allergic reaction attributed to a hospital bed cover. The distribution of adverse events was similar between the groups, and all were resolved.
Cömert Kılıç (2021)	No complications related to the IAIs were observed during treatment or follow‐up periods.
Cömert Kılıç and Güngörmüş (2016a)	Some adverse events were observed in four of the 14 patients in the prolotherapy group. Paresthesia spreading to the zygomatic arch and pre‐auricular area was observed in three patients, which resolved over the course of a month with prescribed medications, including vitamin B. A transient blepharospasm occurred in one patient, which recovered after a few weeks. No other complications were observed during treatment and follow‐up periods.
Cömert Kılıç and Güngörmüş (2016b)	No complications occurred during or after these procedures.
Cömert Kılıç et al. (2015)	No complications related to IAIs were observed during the treatment and follow‐up periods.
Dasukil et al. (2022)	All three groups had positive results after treatment, with no significant postoperative complications. Twelve patients reported transient facial paralysis due to local anesthesia (two in Group A, six in Group B, and four in Group C), which was resolved within 1 h.
Fernández‐Ferro et al. (2017)	No complications or adverse events as a result of the procedure or during the follow‐up period were observed.
Guarda‐Nardini et al. (2015)	No adverse events were observed in any of the patients.
Haggag et al. (2022)	Not reported.
Hegab et al. (2015)	Pain and discomfort were associated with PRP injection. Pain was observed during the IAIs and in the following days (during the acute inflammatory phase).
Hepguler et al. (2002)	Not reported.
Huddleston Slater et al. (2012)	Due to the small sample sizes, this study was also unable to identify any side effects of dexamethasone.
Isacsson et al. (2019)	The methylprednisolone group had more adverse events, likely or definitely related to the intervention, mainly increased pain (33%).
Işık et al. (2022)	No perioperative or postoperative adverse events resulting from treatment were recorded.
Işık et al. (2023)	No perioperative or postoperative complications were observed in the groups.
Karadayi and Gursoytrak (2021)	Not reported.
Korkmaz et al. (2016)	No complications or adverse events related to HA IAI or after injection were observed in any patient.
Mustafa et al. (2018)	All patients tolerated prolotherapy IAI well. Two patients experienced transient facial paralysis, which was resolved within 2 h. Postoperative pain was reported by most patients but subsided within 3 to 4 days with paracetamol. Some patients experienced momentary closure of the mouth in a protrusive position, especially those who received 30% Dextrose.
Olsen‐Bergem and Bjør nland (2014)	Not reported.
Ozdamar et al. (2017)	Not reported.
Patel et al. (2016)	Immediately postoperatively, patients experienced tenderness, swelling in the treated TMJ, mild bite alteration, and occasionally slight hearing loss, all of which resolved within a few days.
Sipahi et al. (2015)	No complications or complaints occurred during or after arthrocentesis or IAI.
Yilmaz et al. (2019)	No complications or adverse events related to the treatment methods were observed in any patient.

### 3.3 VAS Scores Quantitative Analysis

Significant reduction in pain intensity (VAS scores) at 6‐month follow‐up comparing to T0 was found, regardless of the IAI used as follows: tenoxicam, a nonsteroidal anti‐inflammatory drug (NSAID), (*p* < 0.00001; *Z* = 10.05; 7.44 [6.28; 8.60]); opioids (morphine and tramadol), (*p* < 0.00001; 5.93 [5.03; 6.83]), HA + PRP, (*p* < 0.00001; 5.10 [4.52; 5.68]); PRP, (*p* < 0.00001; *I* ^2^ = 93%; 4.99 [3.13; 6.85]); HA, (*p* < 0.00001; *I* ^2^ = 84%; 4.85 [3.99; 5.71]); RL, (*p* < 0.00001; *I* ^2^ = 6%; 4.84 [4.39; 5.29]); and dextrose 10% (*p* < 0.00001; 3.07 [2.47; 3.67]), (Figure 3).

Meta‐analysis (Figure 3) indicates that tenoxicam IAI showed the highest mean differences for pain relief, followed by opioids (morphine and tramadol) and the combination of HA with PRP. Despite the moderate effectiveness observed for isolated HA and RL, these treatments were surpassed by PRP and opioids. Dextrose 10% showed the lowest mean differences among the options analyzed, regardless of whether the IAI was unguided (US‐: 2.91 [2.22; 3.60]) or ultrasound‐guided (US+: 3.57 [2.36; 4.78]). The heterogeneity between the studies considering the number of HA IAI was low for the tenoxicam, opioids, dextrose 10%, and RL, while variability was higher for PRP and HA. The overall effects were significant (*p* < 0.00001). Moreover, Egger's test showed no evidence of publication bias for the VAS outcome (*t* = −0.21, df = 15, *p* = 0.838), with the corresponding funnel plot presented in Figure 7a.

Meta‐analysis considering VAS scores using HA IAI showed that both one and two injections were associated with pain reduction (Figure 4). A single injection showed slightly greater effectiveness (4.95 [3.33; 6.58]) compared to two injections (4.71 [3.58; 5.84]), with no statistically significant difference (*p* = 0.81, *I* ^2^ = 0%). From a practical standpoint, the advantage of administering one or two injections appears to be minimal, regardless of whether HA is used alone or alongside arthrocentesis. Similarly, a study by (Korkmaz et al. 2016) found only a small difference in pain reduction between one HA IAI (4.24 [3.02; 5.46]) and two HA IAIs (4.07 [3.21; 4.93]), which also did not reach statistical significance.

### 3.4 MMO Quantitative Analysis

**FIGURE 5:**
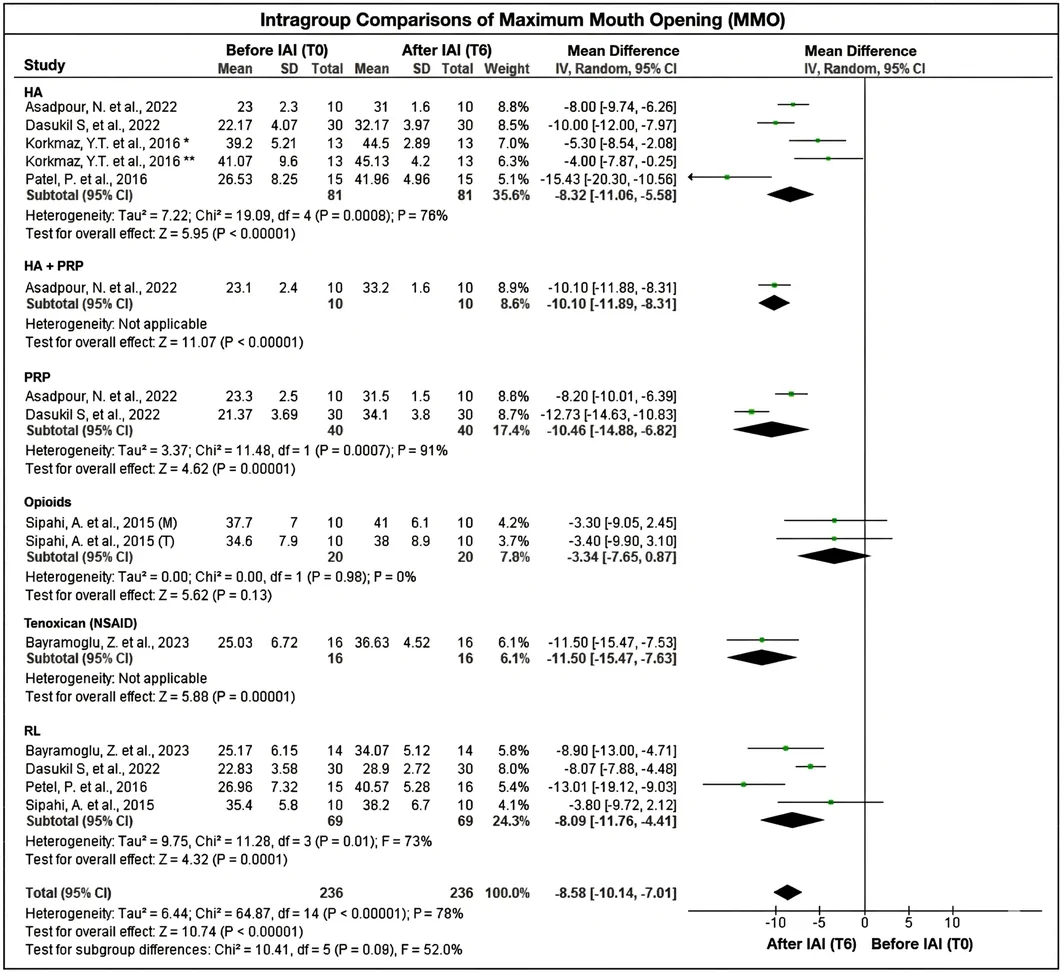
Meta‐analysis of intragroup comparisons of maximum mouth opening (MMO) in the studies included in the meta‐analysis, measured at baseline (T0) and 6 months after (T6) intra‐articular injection (IAI). Mean difference values reflect the increase in MMO in all groups, except for the opioid group. (*), one injection of HA; (**), two injections of HA; HA + PRP, hyaluronic acid combined with platelet‐rich plasma; HA, hyaluronic acid; IAI, intra‐articular injection; MMO, maximum mouth opening; NSAID, tenoxicam; opioids, tramadol (T) and morphine (M); PRP, platelet‐rich plasma; RL, Ringer's lactate; T0, baseline; T6, 6 months after the IAI.

**FIGURE 6:**
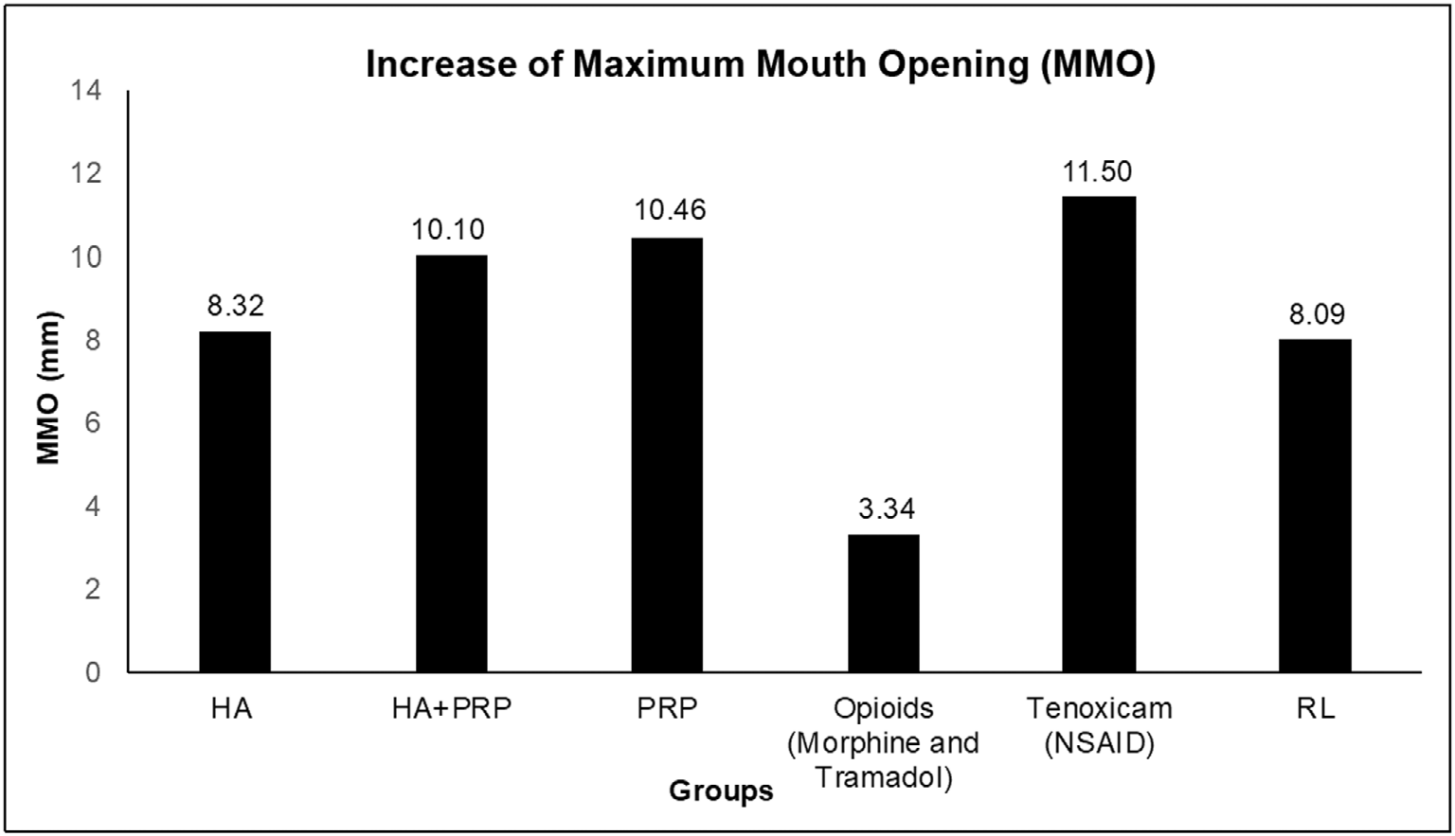
Increase in maximum mouth opening (MMO) following intra‐articular injection (IAI) procedures. The *Y*‐axis represents the gain in MMO (in millimeters), while the *X*‐axis displays the substances tested. Substances are listed in order of greatest to least MMO gain: Tenoxicam (NSAID); PRP (platelet‐rich plasma); HA + PRP (hyaluronic acid combined with platelet‐rich plasma); HA (hyaluronic acid); RL (Ringer's lactate); and opioids (morphine and tramadol), which showed the smallest increase and were ineffective in improving MMO.

Meta‐analysis considering MMO showed that opioids failed to significantly increase MMO (*p* = 0.13) (Figure 5). All other IAI analyzed were effective in improving MMO. Tenoxicam produced the greatest increase (+11.50 mm). PRP also led to a substantial improvement (+10.46 mm), although the analysis showed high inconsistency (*I* ^2^ = 91%) and significant heterogeneity (*p* < 0.05), indicating considerable variability among the included studies (Figure 6).

**FIGURE 7:**
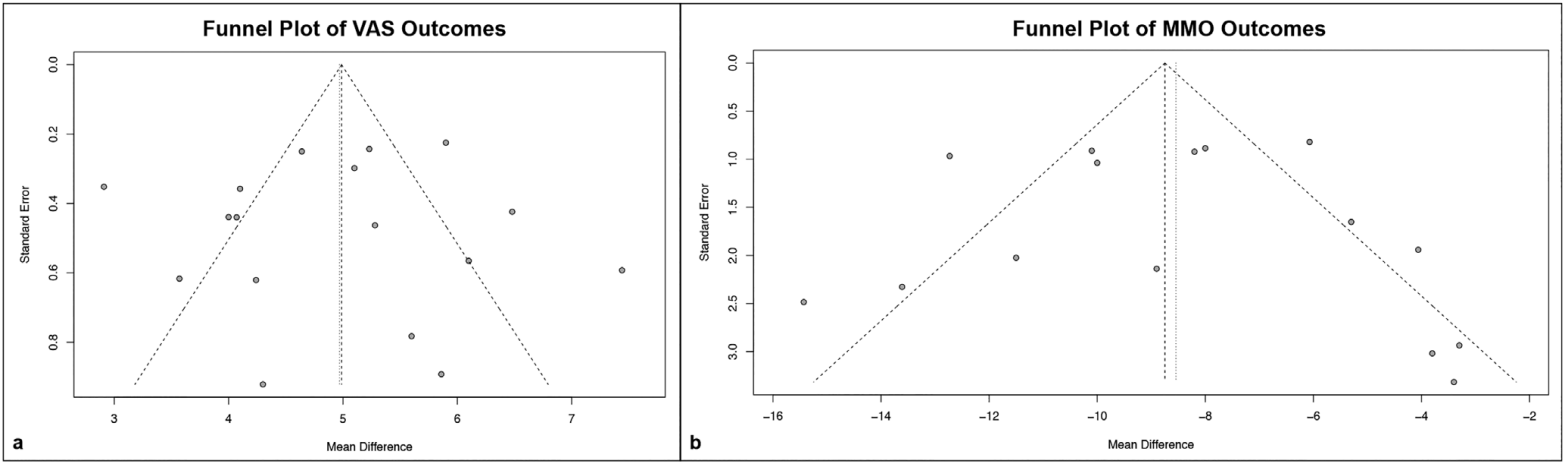
Funnel plots for the assessment of publication bias in (a) Visual Analog Scale (VAS) and (b) Maximum Mouth Opening (MMO) outcomes, generated in R (version 4.5.1; R Core Team, Austria).

The combination of HA with PRP resulted in a +10.10 mm improvement in MMO, while HA alone led to a +8.32 mm. Both interventions were associated with high inconsistency (*I* ^2^ = 79%) and significant heterogeneity (*p* < 0.05), reflecting substantial variability across studies. RL produced a +8.09 mm increase in MMO, also accompanied by significant heterogeneity (*p* < 0.05; *I* ^2^ = 73%), indicating similar variability. Opioids (morphine and tramadol) showed the smallest improvement, with a +3.34 mm increase. Besides, Egger's test showed no evidence of publication bias for the MMO outcome (*t* = 0.34, df = 13, *p* = 0.736), with the corresponding funnel plot presented in Figure 7b.

When considering confidence intervals, PRP had the widest range (8.87), suggesting greater variability in treatment outcomes. This was followed by opioids (8.62), tenoxicam (7.94), RL (7.35), and HA (5.48). Notably, the combination of HA with PRP had the narrowest confidence interval (3.58), indicating more consistent results than the other treatments. Tenoxicam demonstrated the highest mean improvement in MMO among all TMJ management options, followed by PRP and the HA + PRP combination. HA and RL also led to meaningful improvement in MMO.

### 3.5 Qualitative Analysis

Twenty‐three were described only qualitatively among the thirty RCTs included in this systematic review according to different IAI as follows:

#### 3.5.1 Hyaluronic Acid

Studies on HA IAI for the TMD suggest that HA alone is effective in reducing pain and increasing MMO, with sustained effects observed up to 6 months of follow‐up (Bertolami et al. 1993; Hepguler et al. 2002). No additional benefits were reported from oral supplementation with glucosamine, chondroitin, and methylsulfonylmethane (GCM) (Cömert Kılıç 2021). When compared to PRP, HA IAI was found to be less effective but still a valid therapeutic option (Hegab et al. 2023). Similarly, PRGF demonstrated slightly greater efficacy than HA (Fernández‐Ferro et al. 2017). Arthrocentesis combined with HA was more effective than PRP IAI (Cömert Kılıç and Güngörmüş 2016a, 2016b). However, some studies have suggested that HA may not be consistently effective when used in combination with arthrocentesis or arthroscopy (Bergstrand et al. 2019; Ozdamar et al. 2017), while one reported greater effectiveness in these combinations compared to HA alone (Fernández‐Ferro et al. 2017). Additionally, a treatment protocol involving five sessions of HA IAI proved to be more effective than a single session (Ayesh et al. 2008; Castaño‐Joaqui et al. 2021; Guarda‐Nardini et al. 2015).

#### 3.5.2 Platelet‐Rich Fibrine (PRF), Plasma Rich in Growth Factors (PRGF), and PRP IAI

The combination of arthrocentesis with PRF has been effective for long‐term pain reduction and improving mandibular function in TMD patients, particularly those classified as Wilkes stage 3, suggesting that PRF may be especially beneficial in more severe cases like Wilkes stage 5 (Işık et al. 2022, 2023; Karadayi and Gursoytrak 2021). Arthrocentesis combined with PRGF IAI was more effective than HA alone in improving MMO and reducing pain (Fernández‐Ferro et al. 2017). Similarly, the combination of arthrocentesis and PRP showed superior results compared to arthrocentesis alone (Fernández‐Ferro et al. 2017). Both PRF and PRP IAI demonstrated better outcomes when preceded by arthrocentesis. However, findings on PRP versus HA are mixed. While one study found PRP more effective for TMJ osteoarthritis (Yilmaz et al. 2019), another reported no superiority over HA, indicating PRP may not be suitable as a first‐line treatment for TMD (Cömert Kılıç and Güngörmüş 2016a, 2016b). However, combining PRP with HA resulted in better outcomes than using either alone (Cömert Kılıç et al. 2015).

#### 3.5.3 Dissociative Anesthetic

A single study (Ayesh et al. 2008) evaluated ketamine, an N‐methyl D‐Aspartate (NMDA), and did not show efficacy in the treatment of TMJ arthralgia, suggesting that peripheral NMDA receptors play a limited role in the pathophysiology of this disorder.

#### 3.5.4 Dextrose

One study (Haggag et al. 2022) highlighted the efficacy of dextrose 25% in pain reduction and improvement of MMO. However, another study (Mustafa et al. 2018) did not show significant differences between concentrations (10%, 20%, or 30%), while another RCT (Cömert Kılıç and Güngörmüş 2016a, 2016b). found no superiority of dextrose IAI over the placebo (saline solution and 2% articaine or mepivacaine).

#### 3.5.5 Corticosteroids (Dexamethasone, Methylprednisolone, and Triamcinolone Hexacetonide)

Dexamethasone (1 mL) did not offer additional improvements after arthrocentesis (Huddleston Slater et al. 2012). Methylprednisolone (Isacsson et al. 2019), a single dose of 1 mL, 40 mg/mL, was even detrimental, increasing pain in some patients. Triamcinolone hexacetonide, 20 mg/mL (Olsen‐Bergem and Bjør nland 2014), also did not show significant benefits. In summary, the addition of corticosteroids to treatments did not demonstrate relevant positive effects in pain reduction or improvement in joint function when compared to isolated arthrocentesis.

#### 3.5.6 Lactated Ringer's Solution in TMJ Arthrocentesis

The use of RL in TMJ arthrocentesis reduced pain and increased MMO, similar to HA (Bergstrand et al. 2019; Castaño‐Joaqui et al. 2021). In one study (Yilmaz et al. 2019), although RL alone provided symptom relief, the combination of arthrocentesis with RL and HA IAI was more effective in improving masticatory efficiency in patients with disc displacement with and without reduction, compared to HA IAI alone. Similarly, the combination of RL with PRF appeared to be potentially more advantageous than the use of RL alone in arthrocentesis (Karadayi and Gursoytrak 2021). Similarly, in another study, the combination of RL and PRP IAI showed superior results (Cömert Kılıç et al. 2015). Therefore, the combination of arthrocentesis with RL and other substances, such as HA, PRF, or PRP, yields superior results, suggesting that arthrocentesis with RL is effective but can be enhanced with the use of IAI of different substances.

#### 3.5.7 TMJ Arthrocentesis With Saline Solution (SS) or IAI Placebo

Twelve studies evaluated saline solution (SS) for TMD treatment. One study found SS had similar effects to ketamine for TMJ arthralgia (Ayesh et al. 2008). Arthrocentesis with SS IAI was more effective than SS with dexamethasone IAI (Huddleston Slater et al. 2012). Indeed, methylprednisolone was not superior to SS for pain reduction (Isacsson et al. 2019). SS IAI showed comparable effects to dextrose in some studies (Cömert Kılıç and Güngörmüş 2016b; Mustafa et al. 2018), while in a more recent study, dextrose was more effective than SS (Haggag et al. 2022). SS was less effective than HA (Bertolami et al. 1993; Hepguler et al. 2002; Ozdamar et al. 2017) and PRGF (Fernández‐Ferro et al. 2017), and showed lower efficacy compared to PRF (Işık et al. 2022, 2023). Overall, SS appears limited in TMD treatment, with other substances showing better results when added. It mainly served as a control or baseline with no significant benefits on its own.

### 3.6 Adverse Events of Intra‐Articular Injections

The adverse events reported in the studies included in the meta‐analysis are summarized in Table 2. Among the 30 evaluated RCTs, 46.67% did not report adverse effects, 23.33% did not provide data, and 30% reported AEs, suggesting a favorable safety profile.

### 3.7 Quality Evidence, GRADEpro

The GRADE assessment revealed that the quality of evidence for the outcome VAS was low for HA, HA + PRP, corticosteroids, and RL; very low for ketamine; and moderate for PRP (Supporting Information S3). For the outcome MMO, the quality of evidence was low for HA, HA + PRP, opioids (morphine and tramadol), and tenoxicam; very low for dextrose; and moderate for PRP (Supporting Information S4). The main issues found were lack of blinding and randomization, potential confounding effects of the intervention, small sample size, unbalanced comparisons between experimental groups for VAS and MMO outcomes, and others.

## 4 Discussion

All IAI assessed reduced pain, with tenoxicam being the most effective, followed by opioids, and the HA and PRP combination. For MMO, only opioids (morphine and tramadol) were ineffective at 6‐month follow‐up, while tenoxicam, PRP, and HA‐PRP showed the best results. About 46.67% of RCTs did not report adverse effects, 23.33% did not provide data, and 30% reported AEs. In this sense, while no serious adverse events were reported, the limited and inconsistent reporting across studies restricts the certainty of any conclusions about safety.

Arthrocentesis combined with IAI was effective for managing painful TMDs and MMO (Ayesh et al. 2008; Bergstrand et al. 2019; Castaño‐Joaqui et al. 2021). IAI with RL or SF improved joint mobility and pain reduction in 86% of patients (Bayramoglu et al. 2023; Dasukil et al. 2022; Marzook et al. 2020; Monteiro et al. 2020; Patel et al. 2016; Sipahi et al. 2015). The isolated use of RL, which has alkalinizing properties, may be effective in severe cases of articular TMD (Aktas et al. 2010a). Meta‐analysis also showed RL efficacy in pain reduction and an increase in MMO, with studies qualitatively assessed also supported these findings (Bayramoglu et al. 2023; Dasukil et al. 2022; Patel et al. 2016; Sipahi et al. 2015). The use of saline solution (SS) IAI as a placebo or irrigating solution results in divergence in its efficacy. Some authors highlight the limitations of SS as an effective TMD treatment, noting the lack of significant therapeutic benefits (Ayesh et al. 2008; Bertolami et al. 1993; Cömert Kılıç and Güngörmüş 2016b; Fernández‐Ferro et al. 2017; Haggag et al. 2022; Hepguler et al. 2002; Huddleston Slater et al. 2012; Isacsson et al. 2019; Işık et al. 2022, 2023; Mustafa et al. 2018; Ozdamar et al. 2017). RL seems to be a preferable option over SS. Three studies using corticosteroid IAI [i.e., dexamethasone (Huddleston Slater et al. 2012), methylprednisolone (Isacsson et al. 2019), and triamcinolone hexacetonide (Olsen‐Bergem and Bjør nland 2014)] had controversial effects on assessed outcomes, influencing vascular response and inflammatory cells, with uncertain efficacy and duration. Dexamethasone's short half‐life (36–72 h) suggests limited prolonged effects (Huddleston Slater et al. 2012). The studies included in this review showed that HA IAI was more effective than dexamethasone with fewer side effects (Neo et al. 1997). Combining corticosteroids with HA yielded better results, but HA alone was superior in pain reduction and increasing MMO (Bjørnland et al. 2007; Liu et al. 2020). A previous study (Olsen‐Bergem and Bjør nland 2014) compared Triamcinolone hexacetonide (20 mg/mL) with arthrocentesis combined with SS and vitamin B12 in juvenile idiopathic arthritis, finding no additional benefits for pain or function. However, triamcinolone was preferred for rheumatoid arthritis due to its slow absorption and prolonged effects (Derendorf et al. 1986). A clinical trial (Isacsson et al. 2019) showed methylprednisolone did not reduce TMJ arthralgia pain and worsened symptoms, while another (Kopp et al. 1991) found it effective with HA for muscle sensitivity and MMO in TMJ rheumatoid arthritis. The divergence among studies highlights the complexity of corticosteroid use in the TMJ, suggesting they may not be the best IAI option. Single corticosteroid injections did not cause detectable radiographic damage, but multiple injections may be harmful (Guarda‐Nardini et al. 2015). Therefore, corticosteroids should be avoided due to their limited efficacy and potential side effects.

Tenoxicam showed superior results in pain reduction and MMO increase, highlighting its advantages over corticosteroids. Tenoxicam IAI showed superior postoperative analgesia in arthroscopies, with fewer side effects and no cartilage destruction, unlike other agents (Colbert et al. 1999). IAI was more effective in TMJ pain relief and inflammation control than oral or intravenous use (Bayramoglu et al. 2023). In our meta‐analysis, tenoxicam IAI showed superior postoperative analgesia in arthroscopies, with fewer side effects and no cartilage destruction, unlike other agents (Aktas et al. 2010b, 2010a). Indeed, it produced the best results for pain reduction (7.44 [6.28; 8.60]) and MMO increase (+11.50 mm), even, the original study concluded that tenoxicam did not outperform isolated arthrocentesis in terms of MMO, pain, and joint sounds. Arthrocentesis, with or without tenoxicam, was effective in pain reduction and increasing MMO, with no significant difference between IAI (Emes et al. 2014; Yapici‐Yavuz et al. 2018). In another study (Gencer et al. 2014), HA was more effective in pain reduction than tenoxicam and betamethasone, with no significant differences between tenoxicam and HA. While it may represent a viable IAI option, its long‐term effects remain uncertain, and any claims of superiority should be interpreted with caution. Additional high‐quality trials are needed to establish their clinical role. Although intra‐articular Tenoxicam showed the largest mean differences for pain reduction and MMO improvement in the present meta‐analysis, these findings should be interpreted with considerable caution as they are derived from a single RCT with a limited sample size and methodological characteristics that differ from other included studies. Consequently, the observed effect represents a single‐study estimate rather than a robust pooled effect. Further well‐designed RCTs with standardized protocols and larger sample sizes are required to confirm the efficacy of intra‐articular tenoxicam and to establish its comparative effectiveness relative to other injectable agents.

Meta‐analysis showed that opioids (morphine and tramadol) provided a minor, statistically insignificant increase in MMO compared to HA, PRP, HA + PRP, tenoxicam, and RL IAI. However, opioids were the second most effective for pain reduction. Morphine IAI was beneficial for 90% of over 500 patients, with significant pain reduction lasting up to a year (Brennan and Ilankovan 2006). A double‐blind trial (Ziegler et al. 2010) confirmed morphine's pain relief, recommending 10 mg doses over 5 mg. Tramadol showed similar analgesic effects to local anesthetics (Tsai et al. 2001) and peripheral analgesic efficacy in humans (Sousa and Ashmawi 2015). Despite their pain‐relieving effects, opioids had a limited impact on MMO increase (+3.34 mm; *p* = 0.13). Although opioids such as morphine and tramadol demonstrated considerable efficacy in pain reduction, their limited effect on functional improvement (MMO) raises concerns regarding their appropriateness as first‐line treatments for TMDs. Moreover, the potential for opioid misuse, dependence, and systemic side effects must be carefully weighed against their short‐term benefits. In contrast, therapies such as HA and PRP not only contribute to pain relief but also offer functional gains and a lower risk profile. These factors suggest that opioids should be reserved for selected cases and used with caution, especially when safer intra‐articular alternatives are available.

Meta‐analysis suggested that the number of HA IAI did not significantly impact effectiveness, aligning with Ferreira et al. (2025). However, previous studies found five HA IAI was more effective than a single (Guarda‐Nardini et al. 2015) emphasizing the complexity of selecting an IAI protocol based on patient response, cost, and clinician experience. Regarding arthrocentesis followed by HA IAI, one study found similar effectiveness with low or medium molecular weight HA for TMJ osteoarthritis symptoms (Guarda‐Nardini, Cadorin, et al. 2012). Another (Guarda‐Nardini, Olivo, et al. 2012) showed greater benefits for patients aged 45–65 with five weekly arthrocenteses followed by HA IAI, suggesting age‐based treatment personalization. Thus, HA is an effective option for TMD management. The meta‐analysis revealed considerable clinical and methodological heterogeneity across the included studies. This may be attributed to variations in participants' age, the inclusion of different TMD subtypes (such as internal derangement or osteoarthritis), and inconsistencies in IAI protocols regarding the type of substance, dosage, number of applications, and whether arthrocentesis was associated. These factors likely influenced the pooled estimates and should be considered when interpreting the clinical significance and generalizability of the results.

The use of autologous blood derivatives IAI in TMJ procedures showed promising results (Asadpour et al. 2022; Cömert Kılıç et al. 2015; Dasukil et al. 2022; Karadayi and Gursoytrak 2021; Hegab et al. 2015; Karadayi and Gursoytrak 2021; Işık et al. 2022; Işık et al. 2023). PRP IAI was more effective than HA for TMJ osteoarthritis (Hegab et al. 2015) or preferable over HA when suitable (Dasukil et al. 2022). However, some reports suggest PRP does not outperform HA alone and should not be first‐line treatment (Cömert Kılıç and Güngörmüş 2016b). The combination of HA and PRP IAI after arthrocentesis shows a synergistic effect and is more effective than isolated injections (Asadpour et al. 2022; Hegab et al. 2023). Additionally, the use of PRF IAI combined with arthrocentesis has proven more effective than isolated treatments (Fernández‐Ferro et al. 2017). PRF IAI enhances pain relief and mandibular mobility (Fernández‐Ferro et al. 2017; Işık et al. 2022, 2023; Karadayi and Gursoytrak 2021). These findings highlight the importance of personalized treatment and therapeutic combinations, such as the combination of HA and PRP with arthrocentesis. Also, autologous blood derivatives may be cost‐effective, carry a lower risk of allergic reactions, and be suitable for patients avoiding synthetic products.

Important to note that prolotherapy with 10% dextrose was the least effective treatment for pain relief in the meta‐analysis, with literature showing varying opinions on its concentration. Some studies suggest concentrations below 10% cause less inflammation, while higher concentrations lead to a more intense response (Cömert Kılıç and Güngörmüş 2016a). However, other reports indicate no significant differences between concentrations, with 10% being sufficient (Mustafa et al. 2018). Despite modest results, prolotherapy with dextrose remains a promising treatment for TMD due to its therapeutic potential and safety, low cost, and availability (Cömert Kılıç and Güngörmüş 2016a).

Minimally invasive TMJ interventions, such as arthrocentesis with or without IAI, were safe, with mild, transient reported AEs. These AEs, like temporary pain, are considered acceptable as long as the benefits outweigh the risks (Dasukil et al. 2022). The included studies had predominantly women (71%) with an average age of 32, aligning with the highest prevalence of TMD between 20 and 40 years (Schiffman et al. 2014). This systematic review has some limitations and strengths. The meta‐analysis' strength lies in using only RCTs, but the evidence quality was low, and the study had limitations, including a 6‐month follow‐up period and the lack of evaluation of articular regeneration and joint sounds. Additionally, the safety profile should be interpreted with caution, as only 30% of the studies reported adverse events, raising concerns about underreporting.

This systematic review focused on painful articular TMD with structural joint involvement (TMJ osteoarthritis and/or internal derangement) to ensure pathophysiological coherence. Within the DC/TMD framework, arthralgia is a symptom‐based diagnosis, whereas these conditions represent structural intra‐articular pathologies (Schiffman et al. 2014); therefore, arthralgia alone does not define the underlying joint disorder. Intra‐articular interventions primarily target structural and inflammatory mechanisms and may not fully explain pain in isolated TMJ arthralgia. Notably, a recent systematic review published in 2025 (Jogigowda et al. 2025) reported modest and inconsistent benefits of intra‐articular injections for TMJ arthralgia, supporting the present focus on structurally defined joint disorders while acknowledging limited generalizability to isolated arthralgia. Future research should include imaging analyses to assess regenerative responses and structural changes, as well as otological symptoms for a more comprehensive understanding of IAI efficacy. Indeed, despite not being included in the present analysis due to eligibility criteria, polydeoxyribonucleotide (PDRN) emerges as a promising biologically active intra‐articular therapy for articular TMD. High‐quality RCTs with standardized diagnostic criteria and adequate follow‐up are urgently needed to define its clinical role and comparative effectiveness (Kim et al. 2025).

## 5 Conclusion

The combination of arthrocentesis with RL IAI effectively reduced pain and increased MMO, showing better results than SS. Among the IAIs analyzed, tenoxicam showed promising results in terms of pain reduction and MMO improvement. HA, administered alone or combined with PRP, was effective but showed enhanced results when combined with arthrocentesis. Autologous blood derivatives frequently demonstrated superior outcomes compared to HA. Regarding HA protocols, both single and double injections reduced pain according to VAS scores, with a single injection showing slightly greater effectiveness.

In contrast, corticosteroid IAI showed controversial efficacy, possibly due to their short intra‐articular half‐life and potential risks, with dextrose yielding the lowest therapeutic effect. Favorable safety profiles with low adverse events were reported for IAI in 60.87% of the included studies. However, given the limited and low‐to‐moderate quality evidence, these findings should be interpreted cautiously. Well‐designed RCTs are needed to confirm comparative effectiveness and long‐term safety.

## Author Contributions

**Mateus Gaya dos Santos:** conceptualization, investigation, writing – original draft, methodology. **Wellington Luiz de Oliveira da Rosa:** supervision, writing – review and editing, conceptualization, methodology, formal analysis. **Cristian Sbardelotto:** writing – review and editing, supervision, writing – original draft. **Júlia Silva Gomes de Araújo:** writing – original draft, investigation, methodology. **Camila da Silva Rodrigues:** supervision, project administration, writing – review and editing, methodology, conceptualization. **Noéli Boscato:** methodology, supervision, data curation, formal analysis, project administration, writing – review and editing, funding acquisition.

## Funding

This study was supported in part by Coordination for the Improvement of Higher Education Personnel (CAPES—Grant/Award Number: Finance Code 001) and by National Council for Scientific and Technological Development (CNPq—Grant/Award Number: Finance Code 303694/2021‐1 and 406004/2024‐2) and FAPERGS: 24/2551‐0001498‐5.

## Ethics Statement

The authors have nothing to report.

## Conflicts of Interest

The authors declare no conflicts of interest.

## Supporting information


**Appendix S1:** odi70284‐sup‐0001‐AppendixS1.docx.

## Data Availability

The data that support the findings of this study are available on request from the corresponding author. The data are not publicly available due to privacy or ethical restrictions.
