# Supplementary material for: Efficacy and Safety of Intra‐Articular Therapies for Temporomandibular Disorders: A Systematic Review and Meta‐Analysis
Source: Oral Dis. 2026 Mar 12;32(7):1828–43. doi: 10.1111/odi.70284 (PMC13590907; doi:10.1111/odi.70284)
Supplement: Supplementary file 1 [file ODI-32-1828-s001.docx]

**Supplementary Material**

**Supplementary Material 1.** Search keys in the different databases

**Medline/PubMed**

(((((Treatment Outcome [Mesh]) OR (Treatment Outcome) OR (Clinical Effectiveness) OR (Treatment Effectiveness)) AND ((Viscosupplementation [Mesh]) OR (Injection*) OR (Injections, Intra-articular))) AND ((Temporomandibular joint [Mesh]) OR (Temporomandibular joint) OR (TMJ) OR (Ear-jaw articulation) OR (Temporomandibular articulation) OR (Joint temporomandibular) OR (Temporomandibular Joints) OR (osteoarthritis [MeSH Terms] OR (Temporomandibular joint osteoarthritis) OR Temporomandibular joint disorders [Mesh]) OR (Temporomandibular Joint Disorders) OR (Temporomandibular joint syndrome) OR (Temporomandibular joint) OR (Ear-jaw articulation) OR (Joint temporomandibular) OR (Orofacial pain) OR (Facial pain [Mesh]) OR (Facial pain) OR (Temporomandibular Joint Dysfunction Syndrome) OR (Temporomandibular Joint Dysfunction Syndrome [Mesh]) OR (Disorders, Temporomandibular Joint) OR (Temporomandibular Joint Disorder) OR (TMJ Disorders) OR (TMJ Disorder) OR (Temporomandibular Disorders) OR (Temporomandibular Disorder) OR (Temporomandibular Joint Diseases) OR (Disease, Temporomandibular Joint) OR (TMJ Diseases) OR (TMJ Disease))) AND ((Clinical Trial) OR (Intervention Study))) AND ((Dentistry [Mesh]) OR (dentistry) OR (odontology)).

**Scopus**

(TITLE-ABS-KEY((Treatment Outcome [Mesh]) OR (Treatment Outcome) OR (Clinical Effectiveness) OR (Treatment Effectiveness)) AND TITLE-ABS-KEY((Viscosupplementation [Mesh]) OR (Injection*) OR (Injections, Intra-articular)) AND TITLE-ABS-KEY((Temporomandibular joint [Mesh]) OR (Temporomandibular joint) OR (TMJ) OR (Ear-jaw articulation) OR (Temporomandibular articulation) OR (Joint temporomandibular) OR (Temporomandibular Joints) OR (osteoarthritis [MeSH Terms] OR (Temporomandibular joint osteoarthritis) OR Temporomandibular joint disorders [Mesh]) OR (Temporomandibular Joint Disorders) OR (Temporomandibular joint syndrome) OR (Temporomandibular joint) OR (Ear-jaw articulation) OR (Joint temporomandibular) OR (Orofacial pain) OR (Facial pain [Mesh]) OR (Facial pain) OR (Temporomandibular Joint Dysfunction Syndrome) OR (Temporomandibular Joint Dysfunction Syndrome [Mesh]) OR (Disorders, Temporomandibular Joint) OR (Temporomandibular Joint Disorder) OR (TMJ Disorders) OR (TMJ Disorder) OR (Temporomandibular Disorders) OR (Temporomandibular Disorder) OR (Temporomandibular Joint Diseases) OR (Disease, Temporomandibular Joint) OR (TMJ Diseases) OR (TMJ Disease)) AND TITLE-ABS-KEY((Clinical Trial) OR (Intervention Study)) AND TITLE-ABS-KEY((Dentistry [Mesh]) OR (dentistry) OR (odontology)))

**Embase (Elsevier)**

((((((((((((((((((((((((((((((((((('treatment'/exp OR treatment) AND ('outcome'/exp OR outcome) AND ('mesh'/exp OR mesh) OR 'treatment'/exp OR treatment) AND ('outcome'/exp OR outcome) OR 'clinical'/exp OR clinical) AND effectiveness OR 'treatment'/exp OR treatment) AND effectiveness AND ('viscosuCpplementation'/exp OR viscosupplementation) AND ('mesh'/exp OR mesh) OR injection* OR 'injections,'/exp OR injections,) AND 'intra articular' AND temporomandibular AND ('joint'/exp OR joint) AND ('mesh'/exp OR mesh) OR temporomandibular) AND ('joint'/exp OR joint) OR tmj OR 'ear jaw') AND ('articulation'/exp OR articulation) OR temporomandibular) AND ('articulation'/exp OR articulation) OR 'joint'/exp OR joint) AND temporomandibular OR temporomandibular) AND ('joints'/exp OR joints) OR 'osteoarthritis'/exp OR osteoarthritis) AND ('mesh'/exp OR mesh) AND terms OR temporomandibular) AND ('joint'/exp OR joint) AND ('osteoarthritis'/exp OR osteoarthritis) OR temporomandibular) AND ('joint'/exp OR joint) AND ('disorders'/exp OR disorders) AND ('mesh'/exp OR mesh) OR temporomandibular) AND ('joint'/exp OR joint) AND ('disorders'/exp OR disorders) OR temporomandibular) AND ('joint'/exp OR joint) AND ('syndrome'/exp OR syndrome) OR temporomandibular) AND ('joint'/exp OR joint) OR 'ear jaw') AND ('articulation'/exp OR articulation) OR 'joint'/exp OR joint) AND temporomandibular OR orofacial) AND ('pain'/exp OR pain) OR facial) AND ('pain'/exp OR pain) AND ('mesh'/exp OR mesh) OR facial) AND ('pain'/exp OR pain) OR temporomandibular) AND ('joint'/exp OR joint) AND dysfunction AND ('syndrome'/exp OR syndrome) OR temporomandibular) AND ('joint'/exp OR joint) AND dysfunction AND ('syndrome'/exp OR syndrome) AND ('mesh'/exp OR mesh) OR 'disorders,'/exp OR disorders,) AND temporomandibular AND ('joint'/exp OR joint) OR temporomandibular) AND ('joint'/exp OR joint) AND ('disorder'/exp OR disorder) OR tmj) AND ('disorders'/exp OR disorders) OR tmj) AND ('disorder'/exp OR disorder) OR temporomandibular) AND ('disorders'/exp OR disorders) OR temporomandibular) AND ('disorder'/exp OR disorder) OR temporomandibular) AND ('joint'/exp OR joint) AND ('diseases'/exp OR diseases) OR 'disease,'/exp OR disease,) AND temporomandibular AND ('joint'/exp OR joint) OR tmj) AND ('diseases'/exp OR diseases) OR tmj) AND ('disease'/exp OR disease) AND ('clinical'/exp OR clinical) AND ('trial'/exp OR trial) OR 'intervention'/exp OR intervention) AND ('study'/exp OR study) AND ('dentistry'/exp OR dentistry) AND ('mesh'/exp OR mesh) OR 'dentistry'/exp OR dentistry OR 'odontology'/exp OR odontology) AND [embase]/lim

**Web of Science**

((((TS=((Treatment Outcome [Mesh]) OR (Treatment Outcome) OR (Clinical Effectiveness) OR (Treatment Effectiveness) )) AND TS=(  (Viscosupplementation [Mesh]) OR (Injection*) OR (Injections, Intra-articular))) AND TS=(  (Temporomandibular joint [Mesh]) OR (Temporomandibular joint) OR (TMJ) OR (Ear-jaw articulation) OR (Temporomandibular articulation) OR (Joint temporomandibular) OR (Temporomandibular Joints) OR (osteoarthritis [MeSH Terms] OR (Temporomandibular joint osteoarthritis) OR Temporomandibular joint disorders [Mesh]) OR (Temporomandibular Joint Disorders) OR (Temporomandibular joint syndrome) OR (Temporomandibular joint) OR (Ear-jaw articulation) OR (Joint temporomandibular) OR (Orofacial pain) OR (Facial pain [Mesh]) OR (Facial pain) OR (Temporomandibular Joint Dysfunction Syndrome) OR (Temporomandibular Joint Dysfunction Syndrome [Mesh]) OR (Disorders, Temporomandibular Joint) OR (Temporomandibular Joint Disorder) OR (TMJ Disorders) OR (TMJ Disorder) OR (Temporomandibular Disorders) OR (Temporomandibular Disorder) OR (Temporomandibular Joint Diseases) OR (Disease, Temporomandibular Joint) OR (TMJ Diseases) OR (TMJ Disease)   )) AND TS=((Clinical Trial) OR (Intervention Study))) AND TS=((Dentistry [Mesh]) OR (dentistry) OR (odontology))

| **Author** | **Follow-up Time** | **Evaluates VAS** | **Evaluates MMO** | **Total Number of Participants** | **Evaluated Groups** | **Participants per Group** |
| --- | --- | --- | --- | --- | --- | --- |
| (AYESH; JENSEN; SVENSSON, 2008) | 24 hours | Y | N | 18 | Group 1: One injection of Ketamine | 18 |
|  |  |  |  |  | Group 2: Saline solution | 16 |
| (BERGSTRAND et al., 2019) | 47 months | Y | Y | 37 | Group AS: Arthrocentesis with RL + AH | 20 |
|  |  |  |  |  | Group A: Arthrocentesis with RL | 17 |
| (BERTOLAMI et al., 1993) | 6 months | Y | N | 121 | Experimental Group: AH dissolved in saline solution | 80 |
|  |  |  |  |  | Placebo Group: Saline solution injection | 41 |
| (CASTAÑO‐JOAQUI et al., 2021) | 12 months | Y | Y | 51 | Control Group: Arthrocentesis with RL | 25 |
|  |  |  |  |  | Test Group: Arthrocentesis with RL + AH | 26 |
| (CÖMERT KILIÇ; GÜNGÖRMÜŞ, 2016a) | 12 months | Y | Y | 26 | Group 1: Placebo; saline solution | 12 |
|  |  |  |  |  | Group 2: Study; 30% Dextrose solution | 14 |

**Supplementary Material 2.** Main characteristics of the studies qualitatively analyzed.

Supplementary Material 2

| **Author** | **Follow-up Time** | **VAS** | **MMO** | **Total Number of Participants** | **Evaluated Groups** | **Participants per Group** |
| --- | --- | --- | --- | --- | --- | --- |
| (CÖMERT KILIÇ; GÜNGÖRMÜŞ, 2016b) | 12 months | Y | Y | 21 | PRP Group: Arthrocentesis with RL + IPRP | 18 |
|  |  |  |  |  | AH Group: Arthrocentesis with RL + AH | 13 |
| (CÖMERT KILIÇ; GÜNGÖRMÜŞ; SÜMBÜLLÜ, 2015) | 12 months | Y | Y | 30 | Control Group 1: Arthrocentesis with RL | 12 |
|  |  |  |  |  | Study Group 2: Arthrocentesis with RL + PRP | 18 |
| (CÖMERT KILIÇ, 2021) | 12 months | Y | Y | 26 | Control Group: Arthrocentesis with RL + HA | 14 |
|  |  |  |  |  | Experimental Group: Arthrocentesis with RL + HA followed by 3 months of Glucosamine, Chondroitin Sulfate, and Methylsulfonylmethane (GCM) supplementation | 12 |
| (FERNÁNDEZ-FERRO et al., 2017) | 8 months | Y | Y | 100 | Group A: Arthrocentesis with saline solution + PRGF | 50 |
|  |  |  |  |  | Group B: Arthrocentesis with saline solution + AH | 50 |
| (GUARDA‐NARDINI et al., 2015) | 6 months | Y | N | 30 | Group A: Arthrocentesis with saline solution + high molecular weight AH (7,000 kDa) | 10 |
|  |  |  |  |  | Group B: Arthrocentesis with saline solution + medium molecular weight AH (1,200 kDa) | 10 |
|  |  |  |  |  | Group C: 5 sessions of Arthrocentesis with saline solution + medium molecular weight AH (1,200 kDa) | 10 |

Supplementary Material 2

| **Author** | **Follow-up Time** | **VAS** | **MMO** | **Total Participants** | **Groups Evaluated** | **Participants per Group** |
| --- | --- | --- | --- | --- | --- | --- |
| (HAGGAG; AL-BELASY; SAID AHMED, 2022) | 6 months | Y | Y | 30 | Group: 25% Dextrose Injection | 15 |
|  |  |  |  |  | Group: Saline Solution Injection | 15 |
| (HEGAB  et al., 2015) | 12 months | Y | Y | 50 | Group I: PRP + Arthrocentesis with RL | 25 |
|  |  |  |  |  | Group II: HA + Arthrocentesis with RL | 25 |
| (HEGAB  et al., 2023) | 12 months | Y | Y | 90 | Group I: Arthrocentesis with Saline Solution + PRP | 30 |
|  |  |  |  |  | Group II (Control): Arthrocentesis + HA | 30 |
|  |  |  |  |  | Group III: Arthrocentesis + PRP+HA Combination | 30 |
| (HEPGULER  et al., 2002) | 6 months | Y | N | 38 | HA Group: HA | 19 |
|  |  |  |  |  | Placebo Group: Saline Solution | 19 |
| (HUDDLESTON SLATER  et al., 2012) | 6 months | Y | Y | 28 | Control Group: Arthrocentesis with Saline Solution + "Placebo Dose" of Saline Solution | 14 |
|  |  |  |  |  | Intervention Group: Arthrocentesis with Saline Solution + Dexamethasone. | 14 |

| **Author** | **Follow-up Time** | **VAS** | **MMO** | **Total Participants** | **Evaluated Groups** | **Participants per Group** |
| --- | --- | --- | --- | --- | --- | --- |
| (ISACSSON  et al., 2019) | 1 month | Y | N | 52 | Group: Saline Solution | 25 |
|  |  |  |  |  | Group: Methylprednisolone | 27 |
| (IŞIK  et al., 2022) | 12 months | Y | Y | 36 | Control Group: Arthrocentesis with saline | 18 |
|  |  |  |  |  | Group I-PRF: Arthrocentesis with saline + I-PRF | 18 |
| (IŞIK  et al., 2023) | 12 months | Y | Y | 76 | Group AC + I-PRF: Arthrocentesis with saline + I-PRF | 38 |
|  |  |  |  |  | Group AC: Arthrocentesis with saline | 38 |
| (KARADAYI; GURSOYTRAK, 2021) | 3 months | Y | Y | 36 | Control Group: Arthrocentesis with LR | 18 |
|  |  |  |  |  | Experimental Group: Arthrocentesis with LR + I-PRF | 18 |
| (MUSTAFA; GÜNGÖRMÜŞ; MOLLAOĞLU, 2018) | 12 months | Y | Y | 37 | Control Group: Saline Solution | 9 |
|  |  |  |  |  | Group Dextrose 10% | 10 |
|  |  |  |  |  | Group Dextrose 20% | 9 |
|  |  |  |  |  | Group Dextrose 30% | 9 |

Supplementary Material 2

Supplementary Material 2

| **Author** | **Follow-up Time** | **VAS** | **MMO** | **Total Participants** | **Evaluated Groups** | **Participants per Group** |
| --- | --- | --- | --- | --- | --- | --- |
| (OLSEN-BERGEM; BJØRNLAND, 2014) | 8 months | Y | Y | 38 | Group A: Arthrocentesis alone (saline solution and vitamin B12) | 17 |
|  |  |  |  |  | Group B: Arthrocentesis with saline solution and vitamin B12 + Triamcinolone Hexacetonide | 21 |
| (OZDAMAR; ALEV; YARAT, 2017) | 3 months | Y | Y | 24 | Group AH: Arthrocentesis with LR + HA | 14 |
|  |  |  |  |  | Group SS: Arthrocentesis with LR + Saline solution | 10 |
| (YILMAZ; KORKMAZ; TUZUNER, 2019) | VAS and MIO up to 6 months; symptom duration up to 12 months | Y | Y | 90 | Group I (Patients with DDwR) | 18 |
|  |  |  |  |  | Ia: Arthrocentesis with LR + HA |  |
|  |  |  |  |  | Ib: Single injection of HA | 18 |
|  |  |  |  |  | Ic: Control (refusal of any treatment) | 9 |
|  |  |  |  |  | Group II (Patients with DDwoR) | 19 |
|  |  |  |  |  | IIa: Arthrocentesis with LR + HA |  |
|  |  |  |  |  | IIb: Single injection of HA | 18 |
|  |  |  |  |  | IIc: Control (refusal of any treatment) | 8 |

**Legend:** HA refers to hyaluronic acid; PRP refers to platelet-rich plasma; I-PRP refers to injectable platelet-rich plasma; HA + PRP refers to hyaluronic acid combined with platelet-rich plasma; RL refers to Ringer’s lactate; DDwR refers to disc displacement with reduction; DDwoR refers to disc displacement without reduction. Y indicates “evaluated” and N indicates “not evaluated.” VAS refers to the Visual Analog Scale; MMO refers to maximum mouth opening.

**Supplementary Material 3. GRADEpro Question:** Was there a reduction in pain, based on VAS scores, in individuals diagnosed with articular temporomandibular disorders (TMD) and adverse effects using arthrocentesis and/or intra-articular injection (IAI), comparing pre-treatment (baseline, T0) and post-treatment (six months, T6) in the studies included in the meta-analysis, as well as other time intervals in studies analyzed qualitatively?

**Context:** VAS analysis for IAI substances in the temporomandibular joint (TMJ).

| **Assessment of Certainty** | | | | | | | **№ of patients** | | **Effect** | | **Certain** |
| --- | --- | --- | --- | --- | --- | --- | --- | --- | --- | --- | --- |
| **№ of studies** | **Study design** | **Risk of bias** | **Inconsistency** | **Evidence indirect** | **Imprecision** | **Other considerations** | **Arthrocentesis and/or IAI with any substance injected into the intra-articular space (IAA)** | **Pre (time zero, T0) and post injection treatment comparisons between minimally invasive procedures (i.e. arthrocentesis and/or IAI)** | **Relative (95% CI)** | **Absolute (95% CI)** |  |
| **HA IAI** | | | | | | | | | | | |
| 16 | randomized clinical trials | Severe^a^ | Not Severe | Not Severe | Severe^b^ | None | 0 | 485 | - | **0**  (0 to 0) | ⨁⨁◯◯ Low^a,b^ |
| **PRP IAI** | | | | | | | | | | | |
| 5 | randomized clinical trials | Not Severe | Not Severe | Not Severe | Severe^c^ | None | 0 | 113 | - | **0**  (0 to 0) | ⨁⨁⨁◯ Moderate^c^ |
| **HA + PRP IAI** | | | | | | | | | | | |
| 2 | randomized clinical trials | Severe^a^ | Not Severe | Not Severe | Severe^c^ | None | 0 | 40 | - | **0**  (0 to 0) | ⨁⨁◯◯ Low^a,c^ |

IAI refers to intra-articular injection; HA refers to hyaluronic acid; PRP refers to platelet-rich plasma; HA + PRP refers to hyaluronic acid combined with platelet-rich plasma; CI refers to confidence interval; VAS refers to Visual Analog Scale; MMO refers to maximum mouth opening.

| **Assessment of Certainty** | | | | | | | **№ of patients** | | **Effect** | | **Certain** |
| --- | --- | --- | --- | --- | --- | --- | --- | --- | --- | --- | --- |
| **№ of studies** | **Study design** | **Risk of bias** | **Inconsistency** | **Evidence indirect** | **Imprecision** | **Other considerations** | **Arthrocentesis and/or IAI with any substance injected into the intra-articular space** | **Pre (time zero, T0) and post injection treatment comparisons between minimally invasive procedures (i.e. arthrocentesis and/or IAI)** | **Relative (95% CI)** | **Absolute (95% CI)** |  |
| **Corticoids IAI** | | | | | | | | | | | |
| 3 | randomized clinical trials | Not Severe | Severe^g^ | Not Severe | Severe^c^ | None | 0 | 62 | - | **0**  (0 to 0) | ⨁⨁◯◯ Low^c,g^ |
| **Ketamine IAI** | | | | | | | | | | | |
| 1 | randomized clinical trials | Severe^i^ | Severe^k^ | Not Severe | Very Severe^m^ | None | 0 | 18 | - | **0**  (0 to 0) | ⨁◯◯◯ Very Low ^i,k,l,m^ |
| **RL IAI** | | | | | | | | | | | |
| 8 | randomized clinical trials | Severe^n^ | Not Severe | Not Severe | Severe^c^ | None | 0 | 141 | - | **0**  (0 to 0) | ⨁⨁◯◯ Low^c,n^ |

IAI refers to intra-articular injection; RL refers to Ringer’s lactate; CI refers to confidence interval; VAS refers to Visual Analog Scale; MMO refers to maximum mouth opening.

| **Assessment of Certainty** | | | | | | | **№ of patients** | | **Effect** | | **Certain** |
| --- | --- | --- | --- | --- | --- | --- | --- | --- | --- | --- | --- |
| **№ of studies** | **Study design** | **Risk of bias** | **Inconsistency** | **Evidence indirect** | **Imprecision** | **Other considerations** | **Arthrocentesis and/or IAI with any substance injected into the intra-articular space** | **Pre (time zero, T0) and post injection treatment comparisons between minimally invasive procedures (i.e. arthrocentesis and/or IAI)** | **Relative (95% CI)** | **Absolute (95% CI)** |  |
| **Saline Solution IAI** | | | | | | | | | | | |
| 13 | randomized clinical trials | Severe^o^ | Severe^p^ | Not Severe | Severe^c^ | None | 0 | 264 | - | **0**  (0 to 0) | ⨁◯◯◯ Very Low^c,o,p^ |
| **PRF, I-PRF or PRGF IAI** | | | | | | | | | | | |
| 5 | randomized clinical trials | Severe^q^ | Not Severe | Not Severe | Severe^c^ | None | 0 | 142 | - | **0**  (0 to 0) | ⨁⨁◯◯ Low ^c,q^ |

IAI refers to intra-articular injection; PRP refers to platelet-rich plasma; PRF refers to platelet-rich fibrin; I-PRF refers to injectable platelet-rich fibrin; PRGF refers to plasma rich in growth factors; CI refers to confidence interval; VAS refers to Visual Analog Scale; MMO refers to maximum mouth opening.

**Explanations of Supplementary Material 3.**

a. Fifty percent of the studies exhibited design flaws, such as lack of randomization or blinding, while the remaining 50% showed no risk of bias in study design.

b. Small sample sizes, wide confidence intervals, absence of confidence intervals, or disproportionate allocation between control and experimental groups.

c. Small sample sizes or wide confidence intervals resulted in imprecise outcomes.

d. All studies exhibited design flaws, including lack of blinding or patient-determined selection of the number of injections.

e. Seventy-five percent of the studies demonstrated inconsistencies due to imprecise results, whereas 25% showed no inconsistencies.

f. Small sample sizes, wide confidence intervals, or absence of confidence intervals.

g. Very small sample sizes reduced the consistency of the results.

h. Small sample sizes (n) and wide confidence intervals overlapping the mean.

i. Study design flaws due to inadequate blinding.

j. Small sample sizes led to uncertainty regarding the effect estimate and imprecision.

k. The generalizability of the results is limited by small sample sizes and the exclusion of male participants, affecting confidence in the evidence for pain relief.

l. Exclusion of male participants compromises the generalizability of the results, leading to indirectness by restricting the evidence to the female population.

m. Analyses included only female patients to avoid bias from male participants, who were excluded; the sample size was considered too small.

n. Sixty-two point five percent of the studies exhibited design flaws, such as inadequate randomization or blinding, while 37.5% showed no risk of bias in study design.

o. Fifty-three point eight-four percent of the studies exhibited design flaws, such as inadequate randomization or blinding, while 46.16% showed no risk of bias in study design.

p. Forty-six point one-five percent of the studies demonstrated severe inconsistency due to imprecise results, high heterogeneity, and small sample sizes; 38.46% showed no inconsistency, while 15.38% exhibited very severe inconsistency due to the exclusion of six patients with elevated total protein values after randomization.

q. Sixty percent of the studies exhibited design flaws, such as lack of blinding or deficiencies in the randomization process.

**Supplementary Material 4.**

**GRADEpro Question:** Was there an increase in maximum mouth opening (MMO) in individuals diagnosed with joint temporomandibular disorders (TMD), along with adverse effects, when treated with arthrocentesis and/or intra-articular injection (IAI)? This comparison was made between pre-treatment (baseline, T0) and post-treatment (six months, T6) in studies included in the meta-analysis, as well as at other time intervals in studies analyzed qualitatively.

**Context:** MMO analysis for IAI substances in the temporomandibular joint (TMJ).

| **Assessment of Certainty** | | | | | | | **№ of patients** | | **Effect** | | **Certain** |
| --- | --- | --- | --- | --- | --- | --- | --- | --- | --- | --- | --- |
| **№ of studies** | **Study design** | **Risk of bias** | **Inconsistency** | **Evidence indirect** | **Imprecision** | **Other considerations** | **Arthrocentesis and/or IAI with any substance injected into the intra-articular space** | **Pre (time zero, T0) and post injection treatment comparisons between minimally invasive procedures (i.e. arthrocentesis and/or IAI)** | **Relative (95% CI)** | **Absolute (95% CI)** |  |
| **Hyaluronic Acid IAI** | | | | | | | | | | | |
| 13 | randomized clinical trials | Severe | Not Severe | Not Severe | Severe^b^ | None | 0 | 356 | - | **0**  (0 to 0) | ⨁⨁◯◯ Low ^a,b^ |
| **PRP IAI** | | | | | | | | | | | |
| 5 | randomized clinical trials | Severe^i^ | Not Severe | Not Severe | Severe^c^ | None | 0 | 113 | - | **0**  (0 to 0) | ⨁⨁⨁◯ Moderate ^c^ |
| **HA + PRP IAI** | | | | | | | | | | | |
| 2 | randomized clinical trials | Severe^d^ | Not Severe | Not Severe | Severe^e^ | None | 0 | 40 | - | **0**  (0 to 0) | ⨁⨁◯◯ Low ^d,e^ |

IAI refers to intra-articular injection; HA refers to hyaluronic acid; PRP refers to platelet-rich plasma; HA + PRP refers to hyaluronic acid combined with platelet-rich plasma; CI refers to confidence interval; VAS refers to Visual Analog Scale; MMO refers to maximum mouth opening.

| **Assessment of Certainty** | | | | | | | **№ of patients** | | **Effect** | | **Certain** |
| --- | --- | --- | --- | --- | --- | --- | --- | --- | --- | --- | --- |
| **№ of studies** | **Study design** | **Risk of bias** | **Inconsistency** | **Evidence indirect** | **Imprecision** | **Other considerations** | **Arthrocentesis and/or IAI with any substance injected into the intra-articular space** | **Pre (time zero, T0) and post injection treatment comparisons between minimally invasive procedures (i.e. arthrocentesis and/or IAI)** | **Relative (95% CI)** | **Absolute (95% CI)** |  |
| **Dextrose IAI** | | | | | | | | | | | |
| 3 | randomized clinical trials | Severe^f^ | Severe^g^ | Not Severe | Severe^h^ | None | 0 | 39 | - | **0**  (0 to 0) | ⨁◯◯◯ Very Low ^f,g,h^ |
| **Opioids (Morphine and Tramadol) IAI** | | | | | | | | | | | |
| 1 | randomized clinical trials | Not Severe | Severe^e^ | Not Severe | Severe^e^ | None | 0 | 20 | - | **0**  (0 to 0) | ⨁⨁◯◯ Low ^e^ |
| **Tenoxicam (NSAID) IAI** | | | | | | | | | | | |
| 1 | randomized clinical trials | Severe^i^ | Not Severe | Not Severe | Severe^e^ | None | 0 | 16 | - | **0**  (0 to 0) | ⨁⨁◯◯ Low ^e,i^ |

IAI refers to intra-articular injection; CI refers to confidence interval; NSAID refers to nonsteroidal anti-inflammatory drug; VAS refers to Visual Analog Scale; MMO refers to maximum mouth opening.

| **Assessment of Certainty** | | | | | | | **№ of patients** | | **Effect** | | **Certain** |
| --- | --- | --- | --- | --- | --- | --- | --- | --- | --- | --- | --- |
| **№ of studies** | **Study design** | **Risk of bias** | **Inconsistency** | **Evidence indirect** | **Imprecision** | **Other considerations** | **Arthrocentesis and/or IAI with any substance injected into the intra-articular space** | **Pre (time zero, T0) and post injection treatment comparisons between minimally invasive procedures (i.e. arthrocentesis and/or IAI)** | **Relative (95% CI)** | **Absolute (95% CI)** |  |
| **Corticoids IAI** | | | | | | | | | | | |
| 2 | randomized clinical trials | Not Severe | Severe ^i^ | Not Severe | Severe^e^ | None | 0 | 35 | - | **0**  (0 to 0) | ⨁⨁◯◯ Low ^e, j^ |
| **RL IAI** | | | | | | | | | | | |
| 8 | randomized clinical trials | Severe^k^ | Not Severe | Not Severe | Severe ^c, e^ | None | 0 | 141 | - | **0**  (0 to 0) | ⨁⨁◯◯ Low ^c,e,k^ |
| **Saline Solution IAI** | | | | | | | | | | | |
| 9 | randomized clinical trials | Severe ^l^ | Severe ^m^ | Not Severe | Severe ^c^ | None | 0 | 163 | - | **0**  (0 to 0) | ⨁◯◯◯ Very Low ^c,l,m^ |

IAI refers to intra-articular injection; CI refers to confidence interval; RL refers to Ringer’s lactate; VAS refers to Visual Analog Scale; MMO refers to maximum mouth opening.

| **Assessment of Certainty** | | | | | | | **№ of patients** | | **Effect** | | **Certain** |
| --- | --- | --- | --- | --- | --- | --- | --- | --- | --- | --- | --- |
| **№ of studies** | **Study design** | **Risk of bias** | **Inconsistency** | **Evidence indirect** | **Imprecision** | **Other considerations** | **Arthrocentesis and/or IAI with any substance injected into the intra-articular space** | **Pre (time zero, T0) and post injection treatment comparisons between minimally invasive procedures (i.e. arthrocentesis and/or IAI)** | **Relative (95% CI)** | **Absolute (95% CI)** |  |
| **PRF, I-PRF or PRGF IAI** | | | | | | | | | | | |
| 5 | randomized clinical trials | Severe^n^ | Severe ^o^ | Not Severe | Severe ^j^ | None | 0 | 142 | - | **0**  (0 to 0) | ⨁◯◯◯ Very Low ^i,n,o^ |

IAI refers to intra-articular injection; PRF refers to platelet-rich fibrin; I-PRF refers to injectable platelet-rich fibrin; PRGF refers to plasma rich in growth factors; CI refers to confidence interval; VAS refers to Visual Analog Scale; MMO refers to maximum mouth opening.

**Explanations of Supplementary Material 4.**

a. 53.85% of the studies had major design flaws, either due to issues with blinding or randomization, while 46.15% showed no flaws indicating risk of bias.

b. Small sample sizes, high heterogeneity, or wide confidence intervals resulted in uncertainty in the effect estimate and imprecision.

c. Small sample sizes or wide confidence intervals resulted in imprecise results.

d. Fifty percent of the studies had a high risk of bias due to flaws in blinding and randomization, while the other 50% showed no risk of bias.

e. Small sample sizes compromised the precision of estimates and increased uncertainty about the observed effect.

f. All studies exhibited design flaws, including inadequate blinding, deficiencies in randomization, or allowing patients to choose the number of injections.

g. Sixty-six point six percent of the studies showed severe inconsistency due to low precision (heterogeneity, small sample size, and wide confidence intervals), while 33.3% showed no inconsistency.

h. Small sample sizes and wide or absent confidence intervals.

i. Design flaws due to inadequate blinding.

j. Very small sample sizes reduced the consistency of the results.

k. Sixty-two point five percent of the studies had a serious risk of bias due to flaws in randomization or inadequate blinding, while 37.5% showed no risk of bias.

l. Fifty-five point five percent of the studies had a serious risk of bias due to flaws in randomization or inadequate blinding, while 44.4% showed no risk of bias.

m. Combining 44.4% of studies with severe inconsistencies and 11.1% with very severe inconsistencies, a total of 55.5% of studies were affected by small sample sizes, high heterogeneity, or wide confidence intervals. In contrast, 44.4% of the studies showed no inconsistencies in their results.

n. Sixty percent of the studies had design flaws, such as lack of blinding or deficiencies in the randomization process.

o. Sixty percent of the studies showed severe inconsistency due to small sample sizes and wide confidence intervals, while 40% showed no inconsistencies.a. 53.85% of the studies had a major flaw in the design, either due to blinding or randomization, while 46.15% had no flaws that would indicate a risk of bias.

b. A small number of participants, high heterogeneity, or wide confidence intervals resulted in uncertainty in the effect estimate and imprecision.

c. A small number of participants or wide confidence intervals resulted in imprecise results.

d. 50% of the studies had a major risk of bias due to flaws in blinding and randomization, while 50% showed no risk of bias.

e. A small sample size compromises the precision of estimates and increases uncertainty about the observed effect.

f. All studies had a design flaw, either due to inadequate blinding, deficiencies in randomization, or patients choosing the number of injections.

g. 66.6% of the studies showed severe inconsistency due to the low precision of results (heterogeneity, small sample size, and wide confidence intervals), while 33.3% showed no inconsistency.

h. A small number of participants; wide confidence intervals or absence of them.

i. A design flaw due to inadequate blinding.

j. The very small sample size reduced the consistency of the results.

k. 62.5% of the studies had a serious risk of bias in the design, either due to flaws in randomization or inadequate blinding, while 37.5% showed no risk of bias.

l. 55.5% of the studies had a serious risk of bias in the design, either due to flaws in randomization or inadequate blinding, while 44.4% showed no risk of bias.

m. Adding up to 44.4% of studies with severe inconsistencies and 11.1% with very severe inconsistencies, we total 55.5% of studies affected by small samples, high heterogeneity, or wide confidence intervals. In contrast, 44.4% of the studies showed no inconsistencies in the results.

n. 60% of the studies had design flaws, such as the absence of blinding or deficiencies in the randomization process.

o. 60% of the studies showed severe inconsistency in the results due to a small sample size and wide confidence intervals, while 40% showed no inconsistencies.
